# Plant Metabolomics as a Tool for Detecting Adulterants in Edible Plant: A Case Study of *Allium ursinum*

**DOI:** 10.3390/metabo12090849

**Published:** 2022-09-09

**Authors:** Stefan Ivanović, Katarina Simić, Stefan Lekić, Milka Jadranin, Ljubodrag Vujisić, Dejan Gođevac

**Affiliations:** 1 Institute of Chemistry, Technology and Metallurgy, National Institute of the Republic of Serbia, University of Belgrade, Njegoševa 12, 11000 Belgrade, Serbia; 2 Faculty of Chemistry, University of Belgrade, 11000 Belgrade, Serbia

**Keywords:** metabolomics, *Allium ursinum*, *Convallaria majalis*, *Arum maculatum*, adulteration

## Abstract

*Allium ursinum* and poisonous adulterants *Convallaria majalis* and *Arum maculatum* were used as a model for detection of adulterants in edible plant. *A. ursinum* samples were spiked with *C. majalis* and *A. maculatum* to mimic adulteration. Metabolomic fingerprinting of all samples was performed using ^1^H NMR spectroscopy, and the resulting data sets were subjected to multivariate data analysis. As a result of this analysis, signals of adulterants were extracted from the data, and the structures of biomarkers of adulteration from partially purified samples were elucidated using 2D NMR and LC-MS techniques. Thus, isovitexin and vicenin II, azetidine-2-carboxylic acid, and trigonelline indicated adulteration of *A. ursinum* samples with *C. majalis*. Isovitexin was also recognized to be an indicator of adulteration of *A. ursinum* with *A. maculatum*. In conclusion, the case study of *A. ursinum* suggested that plant metabolomics approach could be utilized for identification of low molecular weight biomarkers of adulteration in edible plants.

## 1. Introduction

*Allium ursinum* (known as wild garlic, ramson or bear’s garlic) was used in this study as a model of an edible plant to detect poisonous adulterants that are potentially present in it. This plant has been widely used in traditional medicine and for eating since ancient times [1].

Food contamination, which can be either accidental or intentional, is an unfortunate act that might have serious consequences for human health. Basically, there are several types of food contaminants such as environmental contaminants, food processing contaminants, presence of adulterants, food additives, and migrants from packaging materials [2]. Adulterations in the global food supply chain are becoming increasingly frequent, and that can reduce the quality and function of genuine food or pose a threat and risk to consumer health [3]. Whether those cases were accidental or intentional or caused by misidentification, such cases must be detected. A prominent case of food poisoning caused by misidentification described a woman from Japan presented with intense vomiting and decreased blood pressure and heart rate after consuming a wild plant that she thought it was *Hosta montana*. The herb was later correctly identified as a toxic *Veratrum album* subsp. *oxysepalum* Hult [4]. Accidental ingestion of *Digitalis purpurea* was reported when two people from Iraq consumed cooked “cabbage” harvested in Edinburgh, believing that it was the same plant they ate in their homeland [5]. Three main causes suggested for the increasing frequency of plant food poisoning due to misidentification [6]. The first one is the lack of botanical knowledge for people living in urban areas whose contacts with natural environments are scarce. Next one is the lack of experience of botanists who can misidentify edible species with poisonous ones that inhabit regions outside of their geographical area of expertise. This may be due to the large morphological similarities that often occur between species. The third main cause is related to the introduction of new species, which are not autochthonous to a particular area. In this way, misidentification is possible among both botanists and laymen [6,7].

The basic problem of traditional methods for detecting food fraud is that they are based on a “target list” approach, where a methodology is developed for a set of molecules that are known in advance before analysis [8,9,10]. Although such methods are suitable for routine monitoring, they often fail to detect peculiar adulterations events. Thus, if one exploits untargeted strategies based on a metabolomics approach, more unexpected fraud cases would be recorded.

The main task of untargeted metabolomics is to simultaneously measure as many metabolites as possible from each sample, thereby actually providing a holistic insight into the sample composition [11]. This top-down strategy analyses the global metabolomics profile, generating large amounts of data processed by high performance bioinformatics tools [12]. ^1^H NMR is a fast and simple technique widely used as a profiling tool in plant metabolomics, such as quality control [13].

In recent decades, *A. ursinum* has attracted the attention of modern medicine due to its various health effects. This led to its increased consumption in the raw state, as well as its use as a dietary supplement [14]. *A. ursinum* leaves share morphological similarities with many other plant species, which can cause potential adulteration on the market. Some poisoning accidents due to *A. ursinum* misidentification with *Colchicum autumnale* and *Convallaria majalis* have been described in the scientific literature [15,16,17,18]. *Convallaria majalis* (Lily of the Valley) and *Arum maculatum* (Adam and Eve, Cuckoo pint) were chosen to be models of poisonous adulterants. Namely, the authors of this manuscript have found that *C. majalis* and *A. maculatum* appeared on the same locality and the same period along with *A. ursinum*. Moreover, all three species share morphological similarities, especially in case of young leaves. Lily of the valley is a poisonous plant widespread in temperate climate of Northern Hemisphere. The plant contains cardiac glycosides whose toxicity is attributed to the inhibition of the enzyme Na+/K+-ATPase. Intoxication can cause sinus tachycardia, heart block, fibrillation, and eventually cardiac arrest [19]. *A. maculatum* is a common woodland plant species widespread across temperate climate of Northern Hemisphere. Although the leaves of *A. maculatum* are used for food preparation in some cultures [20], allergic reactions are possible after consumption of any part of the plant [21]. The fruits of this plant, which contain oxalates and saponins, are especially poisonous [22].

This study has three main aims. Initially to set up a relevant pool of samples as a valid foundation for the forthcoming activities: authentic samples of *A. ursinum* and simulated fraudulent samples. In addition, *A. ursinum* will be blended with *C. majalis* and *A. maculatum* to mimic adulteration. The next aim is to perform metabolomics fingerprinting using NMR spectroscopy. The big datasets obtained after fingerprinting will be handled through multivariate data analysis models for distinguishing between authentic and simulated fraudulent samples. The last aim is to determine the structures of biomarkers of adulteration from partially purified samples using 2D NMR and LC-MS techniques.

## 2. Materials and Methods

### 2.1. Plant Sample Procurement

The leaves of *A. ursinum* were harvested in six different locations in Serbia (Košutnjak, in March 2021; Leskovac, in May 2022; Jablanik, in May 2022; Veliko Selo, in May 2022; Višnjica, in May 2022; and Fruška Gora, in March 2021). Each leaf of *A. ursinum* was harvested from a different individual and represents one biological replicate. The leaves of *C. majalis* and *A. maculatum* were harvested in Košutnjak, in May 2022. The samples were identified by Prof. Marjan Niketić, and voucher specimens (17,827, 17,828, 17,829, 17,830, 17,831, 17,832, 17,828, 17,835, 17,836) were retained at the Herbarium of Institute of Botany and Botanical Garden “Jevremovac”, University of Belgrade.

### 2.2. Sample Preparation for NMR Fingerprinting

In this case, 14 biological replicates from each of the six *A. ursinum* collections, along with samples of *C. majalis* and *A. maculatum* were air-dried and ground. Two replicates from each collection of *A. ursinum* were mixed with *C. majalis* or *A. maculatum*, to obtain binary mixtures at 3 different adulteration levels in weight ratio of 10, 30, and 50%. This resulted in a total of 72 simulated fraudulent samples, while 12 samples remained intact, representing a group of authentic samples. All the samples were prepared for NMR measurements according to the instructions from the literature, with modifications [23]. Each sample (40 mg) was extracted with 800 µL of mixture (1:1) containing equal amounts of 90 mM phosphate buffer (pH 6.0) prepared in D_2_O (containing 0.1% TSP-*d_4_*) and MeOH-*d*_4_. The mixtures were sonicated 20 min in an ultrasonic bath (Sonorex Super RK 100, Bandelin, Berlin, Germany). After centrifugation, for 10 min at 15,000 rpm (DLAB D2012 plus, Beijing, China), resulting supernatants were transferred into NMR tubes.

### 2.3. NMR Analysis

A Bruker Avance III 500 NMR spectrometer, equipped with a 5 mm BBI probehead was used to record ^1^H NMR spectra for metabolomics fingerprinting. The signal of TSP-*d*_4_ was used for referencing. Presaturation pulse program with 128 scans, spectral width of 11 ppm, and relaxation delay of 2 s was used for the ^1^H NMR spectra.

^1^H NMR and 2D NMR experiments for the analysis of biomarkers were recorded on the same device and probehead as for metabolomics fingerprinting. Standard pulse sequences for COSY, NOESY, 2D *J*-resolved, HSQC, and HMBC experiments were used.

TopSpin software (version 3.2, Bruker Biospin, Rheinstetten, Germany) was used for the 1D and 2D NMR spectra processing.

### 2.4. Multivariate Data Analysis

After the ^1^H NMR spectra were referenced to the signal of TSP-*d*_4,_ phased and baseline corrected, the regions of δ 0.54–9.50 were binned to integrated regions of equal width (0.04 ppm) using MestReNova software version 6.0.2 (Mestrelab Research, Santiago de Compostela, Spain). SIMCA software (version 17, Sartorius Stedim Biotech, Goettingen, Germany) was used for multivariate data analysis. The NMR data were normalized to the total area, mean centered, and scaled to unit variance. The regions of δ 3.30–3.38 and 4.66–4.90 were excluded from the analysis to avoid areas of the residual MeOH-*d_4_* signal and HOD, respectively.

### 2.5. Purification of Biomarkers from Convallaria majalis

In order to identify metabolites of interest, NMR-guided purification of *C. majalis* was carried out. Dried and ground leaves of *C. majalis* (32 g) were extracted with methanol (3 × 200 mL, 30 min) at 25 °C using an ultrasonic bath. After the methanolic extracts were combined and filtered, solvent was evaporated obtaining 4.97 g of the residue. This was suspended in 100 mL of water and extracted four times with 50 mL of dichloromethane. The remaining aqueous extract was re-extracted with 4 × 50 mL of *n*-butanol. The yield of butanol extract was 0.65 g and aqueous 1.40 g after the solvents were removed under reduced pressure. Using 1D, 2D NMR experiments, and MS data, isovitexin and vicenin II were identified from the butanolic extract.

A portion of the aqueous extract (0.70 g) was fractionated using dry-column flash chromatography using a combination of isocratic and gradient elution on a 21 × 3.4 cm silica gel column. For fractions F1-F44, CHCl_3_/MeOH/H_2_O = 60/34/6 solvent system was used for the elution. Next, for fractions F45-F60 and F61-F66 polarity of the eluent was increased using CHCl_3_/MeOH/H_2_O = 51/42/7 solvent system and MeOH, respectively. A total of 66 initial fractions (20 mL) were collected. Simultaneously with the separation, analytical thin layer chromatography (TLC) was used to combine the fractions based on the same or similar Rf values. Azetidine-2-carboxylic acid was identified in fraction 48.

After solvent was evaporated on a rotary vacuum evaporator from the combined fractions F64-F66, 53.8mg of dried product was obtained where trigonelline was identified.

### 2.6. Purification of Biomarkers from Arum maculatum

Dried and ground leaves of *A. maculatum* (5.3 g) were extracted with methanol (3 × 40 mL, 30min) at 25 °C using an ultrasonic bath. The combined methanolic extracts were filtered, and solvent was removed to give 1.1 g of the residue. This was suspended in 20 mL of water and extracted four times with 10 mL of dichloromethane. The remaining aqueous extract was re-extracted with *n*-buthanol (4 × 10 mL). The yield of *n*-butanol extract was 0.82 g and aqueous 0.18 g after the solvents were removed under reduced pressure. Then, the entire amount of the *n*-buthanol extract was dissolved in methanol and fractionated on a Sephadex LH-20 column measuring 30 × 5.5 cm. Elution was performed with pure methanol to give 35 fractions, 2 mL each. The CHCl_3_/MeOH/H_2_O = 60/30/3 system was used as eluent for TLC. Fraction 31 was identified to be of interest, based on recorded ^1^H NMR spectra. Using 1D and 2D NMR experiments isovitexin was identified in this fraction. The structure of isovitexin was confirmed by standard addition experiment. Isovitexin standard was added into the previously recorded NMR sample and ^1^H NMR was recorded again. Signals of isovitexin completely overlap with signals in original spectrum and intensity of those signals arise comparing to other signals.

### 2.7. LC-MS Analysis of Biomarkers

Acetonitrile (LiChrosolv^®^, hypergrade for LC-MS, Merck, Darmstadt, Germany) and deionized water (18.2 MΩ cm^−1^, Barnstead™ Smart2Pure™ Water Purification System, Thermo Scientific, Waltham, MA, USA) were used for the preparation of the mobile phases for LC-MS analyses.

Ammonium formate was used to prepare the eluent additive (puriss. p.a., eluent additive for LC-MS, Fluka, Ronkonkoma, NY, USA).

The reference solution contained 2.5 mM hexakis (1H,1H,3H-tetrafluoropropoxy)phosphazine, 5 mM purine, and 100 mM ammonium trifluoroacetate (Agilent Technologies, Waldbronn, Germany).

For identification of biomarkers, prepared samples were injected into liquid chromatograph (1290 Infinity LC system; Agilent Technologies, Waldbronn, Germany) consisted of a quaternary pump, an autosampler, a column oven, and a Diode Array Detector (DAD), connected to the Quadrupole Time-of-Flight mass detector (6550 iFunnel Q-TOF MS, Agilent Technologies; Santa Clara, CA, USA). Q-TOF MS was equipped with a dual spray electrospray ion source. Separation of compounds was performed using a Zorbax Eclipse Plus C18 column (100 × 2.1 mm, 1.8 μm, Agilent Technologies). Mobile phase was composed of solvents A (0.1% formic acid and 5 mM ammonium formate in water) and B (0.1% formic acid in acetonitrile). The following gradient program was used: 0–2 min 5% B, 2–12 min 5–95% B, 12–15 min 95% B, 5 min 5% B, at flow rate of 0.40 mLmin^−1^ and the column temperature of 50 °C. The injection volumes were 1 and 2 μL for positive and negative ion modes, respectively. After separation, the compounds were analyzed using both, DAD and mass detectors. DAD detector recorded UV spectra in the range 190–450 nm, and registered chromatograms at 270 and 340 nm. Mass detector recorded data in positive and negative ion modes. The *m*/*z* range was 100–1500, capillary voltage 3500 V, fragmentor voltage 140 V, nozzle voltage 1000 V, skimmer 1 65 V, octupole RF peak 750 V, nitrogen as a desolvatation gas at 200 °C and 14 L min^−1^, nebulizer 35 psig, nitrogen as a sheath gas at 350 °C and 11 L min^−1^, and with no applied collision energy. Additionally, MS/MS spectra were recorded in the same mode using collision energies 20 V and 40 V. For the data acquisition and processing MassHunter software (revisions B.06.01 and B.07.00) was used (Agilent Technologies; Santa Clara, CA, USA).

## 3. Results and Discussion

### 3.1. Metabolomics Fingerprinting

**Figure 1:**
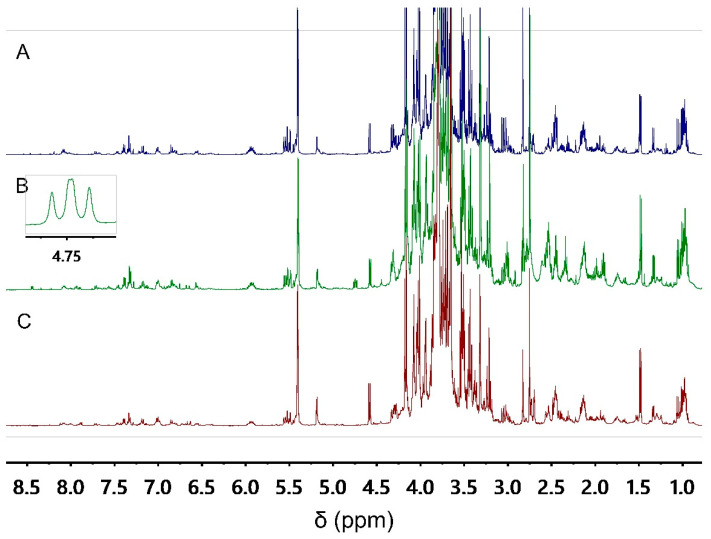
Representative ^1^H NMR spectra of the *A. ursinum* extracts: authentic sample (**A**), spiked with *C. majalis* (**B**), spiked with *A. maculatum* (**C**).

Since metabolite composition of a plant sample can vary according to geographical and ecological factors, variation in tissue composition, multiple biological replicates comprising diverse botanical populations is needed. In this study, we used *A. ursinum* samples collected from six different locations. From each collection, 14 biological replicates were used for fingerprinting. Two of them were analyzed intact, representing a group of authentic samples. Simulated fraudulent samples were obtained by spiking six replicates of *A. ursinum* with *A. maculatum* and more six replicates of *A. ursinum* with *C. majalis*. The simulated fraudulent samples were prepared at 3 different adulteration levels. This together makes a total of 84 samples, which were fingerprinted using ^1^H NMR spectroscopy. Since untargeted metabolomics involves measuring as many metabolites as possible at the same time, a critical step is their extraction. For this occasion, a mixture of aqueous phosphate buffer at pH 6.0 and methanol was used in an equal ratio. This mixture provides a good choice for the extraction of both secondary and primary metabolites [12]. The representative ^1^H NMR spectra of authentic and simulated fraudulent *A. ursinum* samples are shown on Figure 1.

By visual inspection of the ^1^H NMR spectra, similar metabolomic patterns were observed in intact *A. ursinum* samples and those spiked with *A. maculatum* and *C. majalis*. Only in case of adulteration with *C. majalis* an apparent signal at δ 4.75 (dd) is observed. Visual inspection of the NMR spectra is based on the human eye’s inborn ability for pattern recognition. However, the complexity of the spectra can lead the scientist to fail to notice all the relevant signals. Three particularly challenging cases for the visual inspection are poor signal-to-noise ratio overlapping multiplets, and signals which are not aligned [24]. Thus, the datasets obtained after fingerprinting are subjected to multivariate data analysis models in the hope of finding more signals that distinguish between authentic and simulated fraudulent samples.

### 3.2. Multivariate Data Analysis

**Figure 2:**
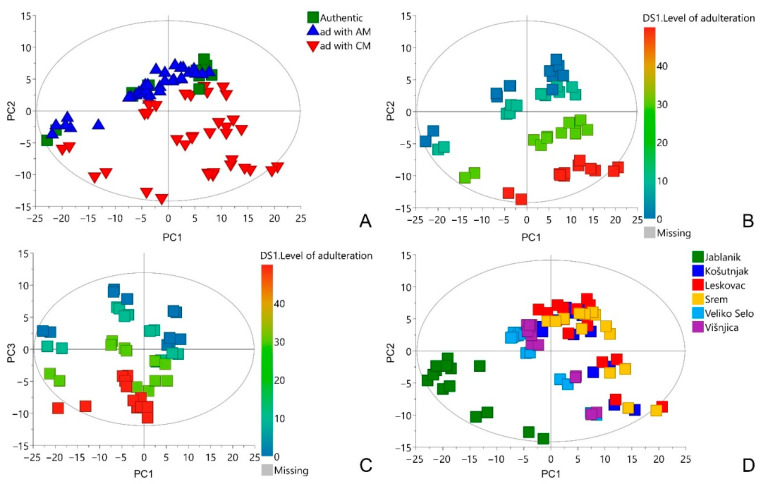
PCA score plots: PC1/PC2 including all studied samples, the scores are colored based on added adulterants. Ad—adulteration, CM—*C. majalis*, AM—*A. maculatum* (**A**); PC1/PC2 including authentic sample and those spiked with *C. majalis*, the scores are colored based on the level of adulteration (**B**); PC1/PC3 including authentic sample and those spiked with *A. maculatum*, the scores are colored based on the level of adulteration (**C**); PC1/PC2 including all studied samples, the scores are colored based on the location of *A. ursinum* harvest (**D**).

The NMR metabolomics fingerprints considering 84 authentic and simulated fraudulent samples of *A. ursinum* were subjected to principal components analysis (PCA). Since PCA is a pattern recognition and unsupervised variable reduction technique, a smaller number of new variables were created that contained most of the variation in the original variables. As a result of this analysis, a model with 10 principal components was obtained, which explains 91.2% of the total variance of the data. According to the PCA score plots of the first two principal components (Figure 2A) no grouping was observed between authentic and simulated fraudulent samples of *A. ursinum* spiked with *A. maculatum*. The samples spiked with *C. majalis* are partially separated from above-mentioned group. The higher the level of adulteration, the greater the separation, which is shown in Figure 2B. Similarly, regarding the level of adulteration, the samples of *A. ursinum* spiked with *A. maculatum* are separated along third principal component, which is shown in the PCA score plots of the first and third principal component (Figure 2C). No strong outliers were observed in any of the score plots. When the score plot is colored according to the location of *A. ursinum* harvest, two separate groups were observed (Figure 2D). This separation may be due to the fact that only a group of samples in the lower left part of the score plot was collected above 1000 m above sea level, while all the others were collected below 300 m. It is known that altitude can affect the chemical composition of many plants [25,26,27]. Based on the aim of this study, it is not crucial what the differences are, but that there is variation in chemical composition in order to create better multivariate models.

After PCA, orthogonal partial least squares on latent structures (OPLS) models were created. In contrast with PCA, in OPLS the novel reduced variables will account for the maximum correlation between NMR metabolomics fingerprints (*X*-variables) and level of adulteration (*Y*-variables). The use of an orthogonal model has the advantage because its interpretation is facilitated *Y* [28]. Therefore, OPLS is suitable for detecting which variables have the highest correlation between NMR metabolomics fingerprints of the studied *A. ursinum* samples and the level of their adulteration.

Two separate OPLS models were created: one including *A. ursinum* adulteration with *C. majalis*, and one *A. ursinum* adulteration with *A. maculatum*. The quality of these models is evaluated by the following parameters: goodness of fit (R^2^) and predictive ability (Q^2^). Predictive ability of the model is estimated by cross validation. The obtained values of R^2^ and Q^2^ close to 1 indicate high goodness of fit and predictive ability of both models (Table 1). However, OPLS models created from metabolomics data can easily be overfitted and their predictability overestimated [29]. Therefore, potential overfitting of the models was checked on three different ways.

Firstly, it was examined whether there is a large discrepancy between the values of R^2^ and Q^2^ and the use of too many components in the models. The difference between R^2^ and Q^2^ was less than 0.01 and the models were created with one predictive and two orthogonal components, indicating no overfitting.

Second, permutation tests were performed to validate the OPLS models. For both models, satisfactory results were obtained (Figures S1 and S2).

Finally, quality of the models was assessed by employing CV-ANOVA. According to the obtained results of this test, the significance of the models was satisfactory with *p* values far below 0.05 (Table 1).

**Figure 3:**
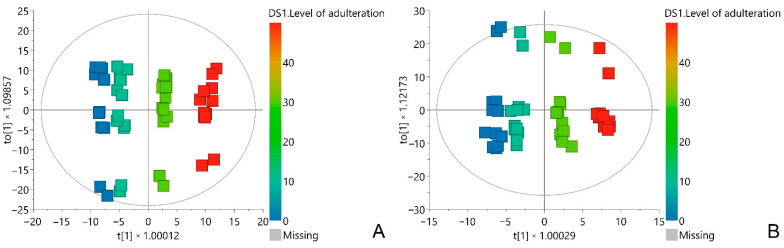
OPLS score plots of the *A. ursinum* sample spiked with *C. majalis* (**A**), and *A. maculatum* (**B**). The scores are colored based on the level of adulteration. t[1]—the first predictive component; to[1]—the first orthogonal component.

By insight into the score plots of the OPLS models, a clear separation of *A. ursinum* samples with lower grades of adulteration from those with higher grades was observed (Figure 3). For the selection of the most influential variables in the OPLS models, the variable influence on projection scores of predictive components (VIP) were used. All VIP score values above 1.5 were considered significant for the correlation. Using the above criteria, it was found that several regions in the loading plots correspond to NMR chemical shifts of biomarkers of adulteration of *A. ursinum* with *C. majalis* and *A. maculatum* (Figures S3 and S4).

### 3.3. Structure Determination of Biomarkers of Adulteration

In order to elucidate the structures of biomarkers whose signals in NMR spectra determined after multivariate data analysis, the extracts of *C. majalis* and *A. maculatum* were partially purified using liquid–liquid extraction and chromatography. At this point, untargeted research strategy is turning into a targeted one. The fractions were not purified completely in order not to waste time and resources. The identities of the adulteration biomarkers were then elucidated after thorough spectral analysis of the partially purified fractions. This was performed using ^1^H and 2D NMR spectra such as COSY, NOESY, homonuclear *J*-resolved, HSQC, and HMBC, as well as HR MS and MS fragmentation data (Figures S5–S27). Thus, the focus in the ^1^H NMR spectra was on the signals that were the variables with the highest VIP score in the OPLS models. The rest of the assignments were performed by careful analysis of 2D NMR spectra. Where appropriate, biomarkers were also identified by their retention times, UV–vis and MS spectral data obtained from LC-DAD-MS (/MS) measurements. All spectral data were then compared with those from literature reports [30,31,32,33,34,35,36,37,38,39].

**Figure 4:**
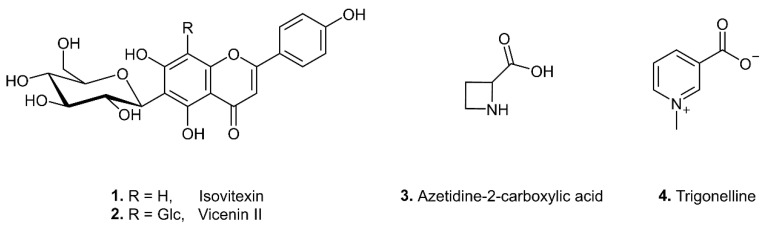
Chemical structures of the biomarkers of adulteration of *A. ursinum* with *C. majalis* (**1**–**4**), and *A. maculatum* (**1**).

The well distinguishable signals in the ^1^H NMR spectra of the *n*-butanol extract of *C. majalis* at δ 7.82 (2H, d, *J* = 8.6 Hz) and 7.91 (2H, d, *J* = 8.4 Hz) gave COSY correlations with the overlapped signals at δ 6.84 (2H, d, *J* = 8.6 Hz) and 6.85 (2H, d, *J* = 8.5 Hz), respectively, indicating the presence of AA′BB′ patterns of two *p*-hydroxyphenyl groups. This was attributed to the B rings of flavonoid *C*-glycosides isovitexin (**1**) and vicenin II (**2**) (Figure 4). Further, isovitexin signal at δ 7.82 (H-2′) gave two NOESY correlations with singlets at δ 6.44 (H-8, A ring) and 6.53 (H-3, C ring), while vicenin II signal at δ 7.91 gave one NOESY correlation with singlet at δ 6.61 (H-3, C ring). Similarly, the aforementioned ^1^H NMR signals of isovitexin (**1**) were also detected in the *n*-butanol extract of *A. maculatum* after purification on Sephadex LH-20 column. The ^1^H NMR signals were also increased upon addition the isovitexin standard to both the *n*-butanol extract of *C. majalis* and the fraction of *A. maculatum*. All NMR data are in agreement with those from the literature [30,31].

Based on the results of the LC-DAD analysis, the *C. majalis n*-butanol extract contains two main components, one at *t*_R_ = 5.27 min vicenin II (**2**) and another at *t*_R_ = 5.69 min isovitexin (**1**), while in the *A. maculatum* extract dominates only one component isovitexin (**1**), *t*_R_ = 5.69 min. Characteristic UV absorbance maxima at 336 and 270 nm indicated flavone or flavonol structure for all these compounds [32].

Overall appearance of MS/MS spectra of corresponding peaks in the LC-MS chromatograms with neutral losses of 90 (C_3_H_6_O_3_), 120 (C_4_H_8_O_4_) and 150 (C_5_H_10_O_5_) mass units, characteristic for the cross-ring cleavages of the saccharide residue, and one to four molecules of water indicated that they are flavone *C*-glycosides (Abad-García et al., 2012; Brito et al., 2014), despite the presence of ions resulting from the losses of hexose (C_6_H_10_O_5_) in both positive and negative modes, characteristic for *O*-glycosides [33].

In the MS spectra of vicenin II, [M+H]^+^ and [M+Na]^+^ ions at *m*/*z* 595.1656 and 617.1476, respectively in positive ionization mode, as well as deprotonated molecular ion [M-H]^−^ at *m*/*z* 593.1522 in negative ionization mode were observed suggesting molecular formula C_27_H_30_O_15_. The characteristic fragment ions at *m*/*z* 503.1204 ([^0,3^X]^−^), 473.1108 ([^0,2^X]^−^), 431.1003 and 311.0573 (the latter two could result from simultaneous cleavage in one and/or both residues of hexose), and at *m*/*z* 577.1550 ([M+H-H_2_O]^+^), 475.1235 ([^0,2^X]^+^), and 455.0942, 437.0843, 433.1129, 415.1022, 397.0918, 379.0814, 367.0812, 337.0706, 313.0706 and 283.0603, arising from simultaneous cleavages in one and/or both hexose residues, appeared as the most intensive in the negative and positive ions MS/MS spectra, respectively of vicenin II, suggesting di-*C*-glycoside. Proposed molecular formula and further pathway reactions, especially presence of the ion at *m*/*z* 271.0609 ([M+H]^+^ ion of apigenin) were in accordance with vicenin II [34,35,36].

Component eluting at *t*_R_ = 5.78 min exhibits molecular formula C_21_H_20_O_10_ according to [M+H]^+^ and [M+Na]^+^ ions at *m*/*z* 433.1125 and 455.0950, respectively in positive ionization mode, as well as deprotonated molecular ion [M-H]^−^ at *m*/*z* 431.0999 in negative ionization mode observed in its MS spectra in both, *C. majalis n*-buthanol extract and fraction of *A. maculatum*.

The main fragment ions observed in the negative ions MS/MS spectrum of this component were at *m*/*z* 413.1468 ([M-H-H2O]^−^), 341.0680 ([^0,3^X]^−^), 311.0571 ([^0,2^X]^−^) and 283.0620 ([^0,2^X-CO]^−^), and at *m/z* 341.0675 ([^0,3^X]^−^), 323.0571 ([^0,3^X-H_2_O]^−^), 311.0569 ([^0,2^X]^−^), 283.0620 ([^0,2^X-CO]^−^) and 269.0458 ([Y_0_]^−^) in the *C. majalis* and *A. maculatum* extracts, respectively. In the MS/MS spectrum of the *C. majalis* the main fragment ions observed were at *m*/*z* 337.0708 ([^0,4^X-2H_2_O]^+^), 313.0710 ([^0,2^X]^+^) and 283.0604 ([^0,1^X]^+^) in positive ions mode. On the other hand, in the *A. maculatum* extract, the main fragment ions observed in the positive ions MS/MS spectrum were at *m/z* 415.1026 ([M+H-H_2_O]^+^), 397.0913 ([M+H-2H_2_O]^+^), 367.0809 ([M+H-2H_2_O-CH_2_O]^+^), 337.0704 ([^0,4^X-2H_2_O]^+^), 313.0706 ([^0,2^X]^+^) and 283.0603 ([^0,1^X]^+^). Despite these differences in the MS/MS spectra of isovitexin in two extracts examined, that could be attributed to the matrix effect, overall fragmentation patterns, together with presence of [M+H-4H_2_O]^+^ ion at *m*/*z* 361,0703 and small intensity ion [Y_0_]^+^ at *m/z* 271,0603, as well absence of [^0,3^X]^+^ ion indicated 6-*C*-glycoside of apigenin [34,35,36,37].

The signal at δ 4.75 (dd), observed also by visual inspection of the ^1^H NMR spectra of *A. ursinum* samples spiked with *C. majalis* was attributed α-proton of azetidine-2-carboxylic acid (**3**) (Figure 4). The two pairs of diastereotopic methylene protons of this cyclic imino acid were assigned according to COSY and HSQC correlations. The NMR data are in agreement with those reported in the literature [38].

Four signals at δ 8.11 (1H, dd, *J* = 8.0, 6.2 Hz), δ 8.86 (1H, br d, *J* = 6.2 Hz), δ 8.88 (1H, br d, *J* = 8.0 Hz) and δ 9.15 (1H, br s) in ^1^H NMR spectra of *A. ursinum* samples spiked with *C. majalis* were assigned to the aromatic protons of the pyridine alkaloid trigonelline (**4**) (Figure 4). A methyl proton singlet at δ H 4.46 showing key HMBC correlations with C-2 and C-6 was also observed in the ^1^H NMR spectrum. This indicated the presence of a methyl group attached to nitrogen and further confirmed the 3-carboxy-1-methylpyridinium cation [39].

### 3.4. Relationships between Biomarkers and Toxic principles in the Adulterants

Flavonoid *C*-glycosides such as isovitexin and vicenin II were found in various medicinal plants and active components of many traditional medicines, exhibiting antiinflammatory, antioxidant, anticancer, antihyperalgesic, and neuroprotective effects [40]. Trigonelline is also found in many plant species and possessing numerous biological activities such as anticancer, antihyperglycemic, cardioprotective, and hepatoprotective [41]. Therefore, biomarkers isovitexin, vicenin II and trigonelline do not represent toxic principles in the studied poisonous adulterants. On the other hand, azetidine-2-carboxylic acid is an antagonist of proline. Incorporation of this cyclic imino acid instead of proline changes the protein folding such as hemoglobin, collagen, and keratin. Its teratogenic effect causing lung and palate malformations in animals has also been demonstrated [42]. Therefore, biomarker azetidine-2-carboxylic acid can be considered as toxic principles in *C. majalis*. It should be emphasized here that cardiac glycosydes are present in this plant as the main toxic ingredients [19]. Their content in dried *C. majalis* leaves vary from 0.1 to 0.5%. As many as 40 different cardiac glycosydes have been identified, in varying proportions. The most abundant among them is convallatoxin, with a ratio of 4 to 40% [43]. In our study, in the ^1^H NMR spectra of the *A. ursinum* samples spiked with *C. majalis* leaves no signals of cardiac glycosydes have been detected. This is not surprising since there are 40 different structures, each with characteristic chemical shifts. The NMR technique is then unable to detect signals of weak intensity in the abundance of much more intense signals of other metabolites.

Although biomarkers isovitexin, vicenin II and trigonelline are not toxic, we suggest that their detectionin *A. ursinum* could indicate adulteration with *C. majalis* containing toxic cardiac glycosydes.

Similarly, isovitexin in *A. ursinum* could indicate adulteration with *A. maculatum*, whose consumption can cause allergic reactions [21].

The principle that the presence of non-toxic metabolites indicates the presence of toxic ones has been also demonstrated in the literature for detection of ricin. Related low mass metabolites from *Ricinus communis* seeds indicated the presence of highly toxic protein ricin [44].

## 4. Conclusions

**Figure 5:**
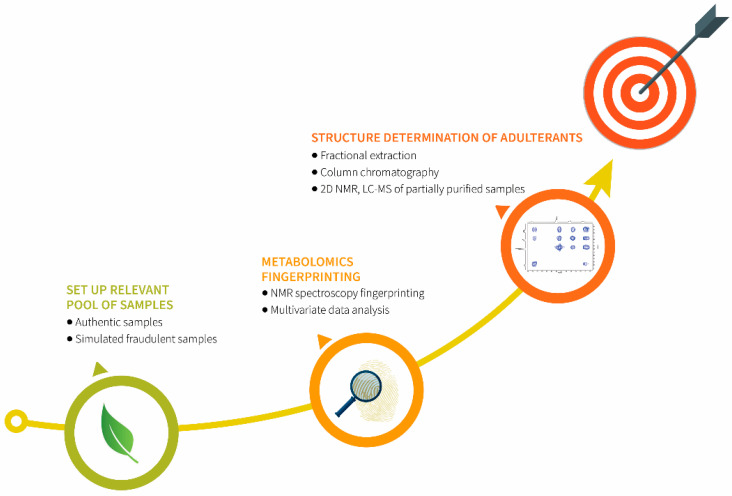
The strategy used for detection of adulterants in *A. ursinum*.

The proposed strategy (Figure 5) on example of *A. ursinum* has proven to be valuable for detecting poisonous adulterants in edible plants. Extraordinarily important for this study was the establishment of a relevant pool of samples as a valid foundation for the forthcoming activities. Furthermore, it was demonstrated that metabolomics fingerprinting using ^1^H NMR spectroscopy combined with multivariate data analysis is a very reliable technique for the detection of *A. ursinum* adulteration. As a result of this analysis, signals of adulterants were extracted from the data. Finally, the structures of biomarkers of adulteration from partially purified samples were elucidated using 2D NMR and LC-MS techniques.

Low sensitivity of NMR somewhat limits its usage to identification of major low molecular weight metabolites in the plant extracts. Nevertheless, NMR offer unique strengths for metabolomics applications thanks to its excellent accuracy and reproducibility, as well as capability to elucidate structures of unknown metabolites. In addition, metabolite profiles can be generated without the need for prior separation [45].

Flavonoid *C*-glycosides isovitexin (**1**) and vicenin II (**2**), cyclic imino acid, azetidine-2-carboxylic acid (**3**), and pyridine alkaloid trigonelline (**4**) indicated adulteration of *A. ursinum* samples with *C. majalis*. Isovitexin (**1**) was also recognized to be an indicator of adulteration of *A. ursinum* with *A. maculatum*.

In conclusion, the case study of *A. ursinum* suggested that plant metabolomics approach could be utilized for identification of low molecular weight biomarkers of adulteration in edible plants.

## Figures and Tables

**Table 1:** Parameters of the PCA and OPLS models.

Model Name	No. of Components	R^2^	Q^2^	*p* (CV-ANOVA)	F (CV-ANOVA)
PCA	10	0.925	0.834	-	-
OPLS, adulteration with CM	1 + 2	0.992	0.988	2 × 10^−37^	543
OPLS, adulteration with AM	1 + 2	0.989	0.981	9 × 10^−34^	357

## Data Availability

Data available on request due to privacy and ethical restrictions.

## Supplementary Materials

The following supporting information can be downloaded at: https://www.mdpi.com/article/10.3390/metabo12090849/s1, Figure S1: Permutation test (N =200) of the OPLS model comprising *A. ursinum* adulterated with *C. Majalis*; Figure S2: Permutation test (N =200) of the OPLS model comprising *A. ursinum* adulterated with *A. maculatum*; Figure S3: Loading plot of the OPLS model comprising *A. ursinum* adulterated with *C.majalis*. The most influential variables with the variable influence on projection (VIP) scores over 1.5 are marked red; Figure S4: Loading plot of the OPLS model comprising *A. ursinum* adulterated with *A.maculatum*. The most influential variables with the variable influence on projection (VIP) scores over 1.5 are marked red; Figure S5: ^1^H NMR spectrum of the n-butanol extract of *C. majalis* containing isovitexin (**1**) and vicenin II (**2**); Figure S6: COSY spectrum of the n-butanol extract of *C. majalis* containing isovitexin (**1**) and vicenin II (**2**); Figure S7: A part of NOESY spectrum of the n-butanol extract of *C. majalis* containing isovitexin (**1**) and vicenin II (**2**); Figure S8: HSQC spectrum of the n-butanol extract of *C. majalis* containing isovitexin (**1**) and vicenin II (**2**); Figure S9: A part of HMBC spectrum of the n-butanol extract of *C. majalis* containing isovitexin (**1**) and vicenin II (**2**); Figure S10: ^1^H NMR spectrum of the fraction of *A. maculatum* containing isovitexin (**1**); Figure S11: ^1^H NMR spectrum of the fraction of *C. majalis* containing azetidine-2-carboxylic acid (**3**); Figure S12: COSY spectrum of the fraction of *C. majalis* containing azetidine-2-carboxylic acid (**3**); Figure S13: Homonuclear J-resolved NMR spectrum of the fraction of *C. majalis* containing azetidine-2-carboxylic acid (**3**); Figure S14: ^1^H NMR spectrum of the fraction of *C. majalis* containing azetidine-2-carboxylic acid (**3**); Figure S15: ^1^H NMR spectrum of the fraction of *C. majalis* containing azetidine-2-carboxylic acid (**3**); Figure S16: ^1^H NMR spectrum of the fraction of *C. majalis* containing trigonelline (**4**); Figure S17: COSY spectrum of the fraction of *C. majalis* containing trigonelline (**4**); Figure S18: HSQC spectrum of the fraction of *C. majalis* containing trigonelline (**4**); Figure S19: HMBC spectrum of the fraction of *C. majalis* containing trigonelline (**4**); Figure S20: LC-DAD-MS analysis of the *C. majalis* extract: **a** LC-DAD (270 nm) chromatogram (4–8 min) and UV spectra of compounds **A** and **B**; **b** and **c** LC-ESI-MS total ion current (TIC) chromatograms (4–8 min) in negative and positive ionization modes, respectively; Figure S21: LC-DAD-MS analysis of the *A. maculatum* extract: **a** LC-DAD (270 nm) chromatogram (4–8 min) and UV spectrum of compound **B**; **b** and **c** LC-ESI-MS total ion current (TIC) chromatograms (4–8 min) in negative and positive ionization modes, respectively.
