# Supplementary material for: Plant Metabolomics as a Tool for Detecting Adulterants in Edible Plant: A Case Study of Allium ursinum
Source: Metabolites. 2022 Sep 9;12(9):849. doi: 10.3390/metabo12090849 (PMC9501555; doi:10.3390/metabo12090849)
Supplement: Supplementary file 1 [file metabolites-12-00849-s001.zip › metabolites-1874606-supplementary.pdf]

Plant metabolomics as a tool for detecting poisonous adulterants in edible plant: a case study of *Allium ursinum*

Stefan Ivanović[1], Katarina Simić[1], Stefan Lekić[2], Milka Jadranin[1], Ljubodrag Vujisić[2] Dejan Gođevac[1]

[1]University of Belgrade - Institute of Chemistry, Technology and Metallurgy, National Institute of the Republic of Serbia, Njegoševa 12, 11000 Belgrade, Serbia

[2]University of Belgrade – Faculty of Chemistry, Belgrade, Serbia

Supplementary Material

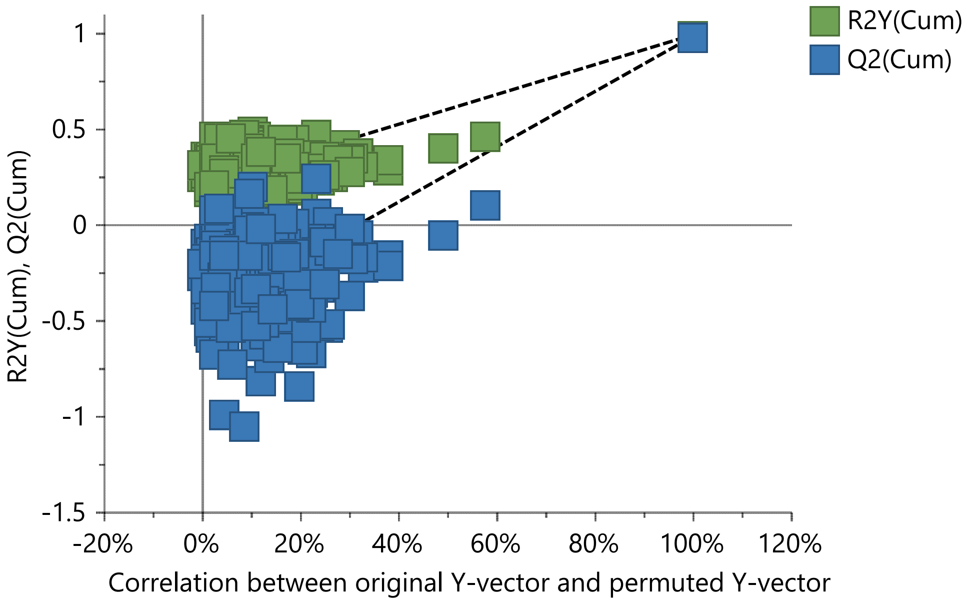

Figure S1. Permutation test (N = 200) of the OPLS model comprising *A. ursinum* adulterated with *C. majalis*

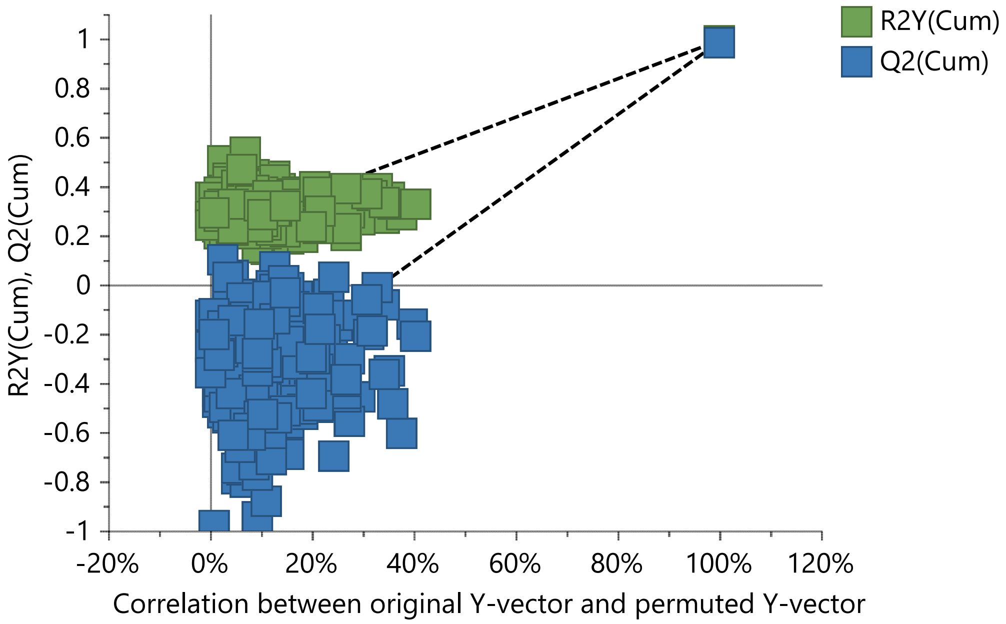

Figure S2. Permutation test (N = 200) of the OPLS model comprising *A. ursinum* adulterated with *A. maculatum*

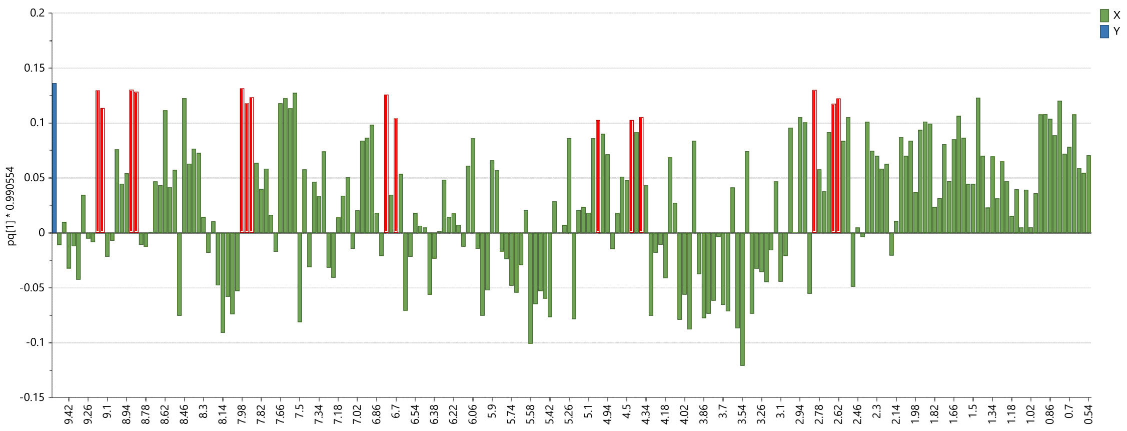

Figure S3. Loading plot of the OPLS model comprising *A. ursinum* adulterated with *C. majalis*. The most influential variables with the variable influence on projection (VIP) scores over 1.5 are marked red.

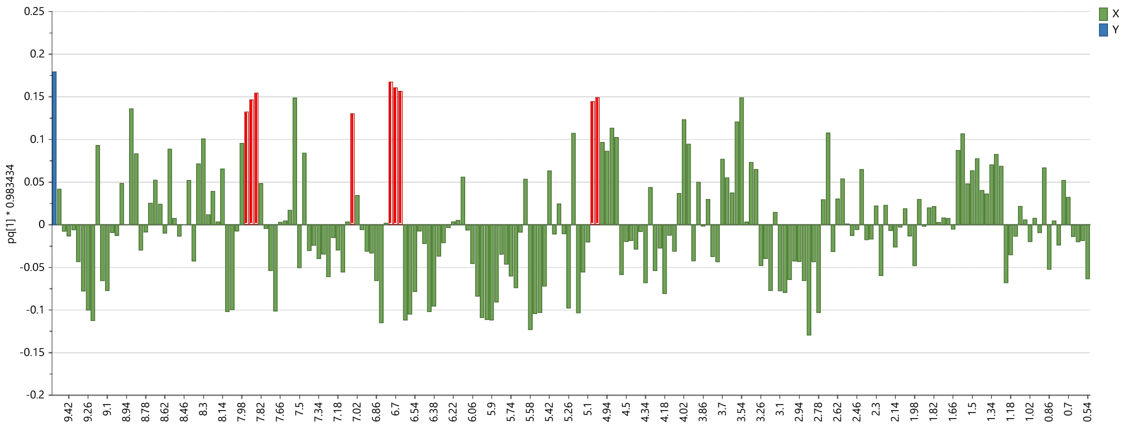

Figure S4. Loading plot of the OPLS model comprising *A. ursinum* adulterated with *A. maculatum*. The most influential variables with the variable influence on projection (VIP) scores over 1.5 are marked red.

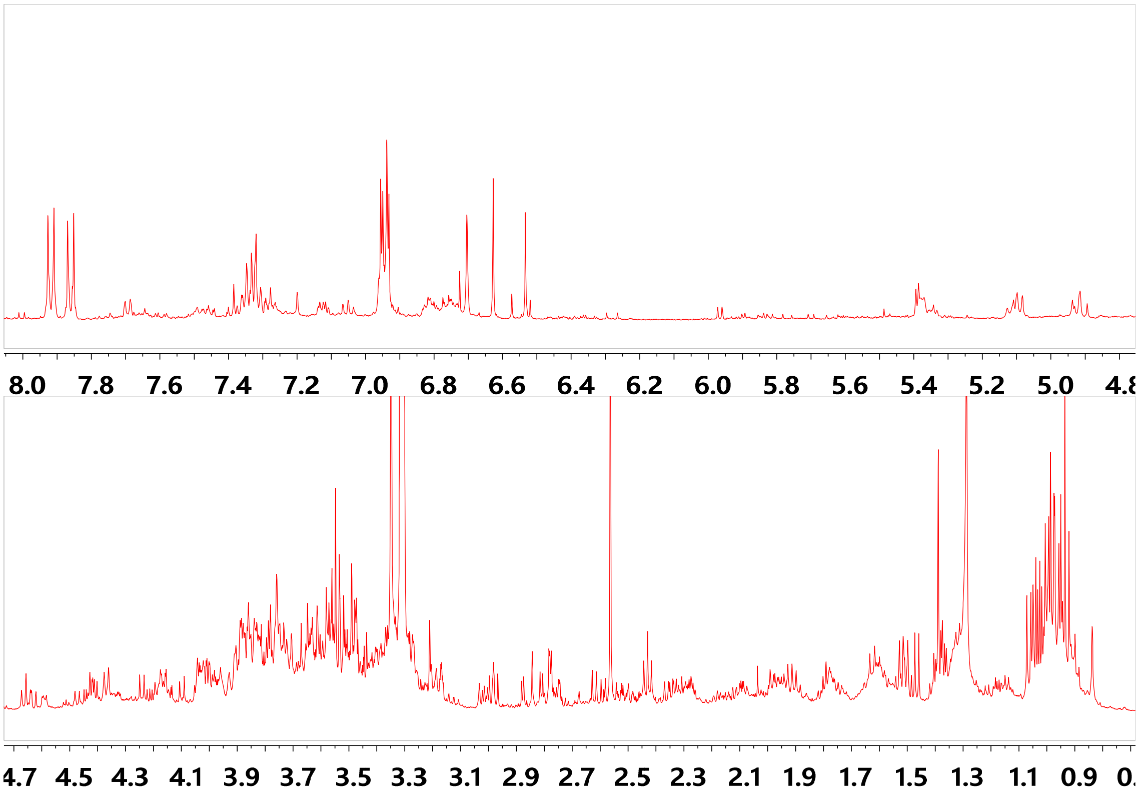

Figure S5. ^{1}H NMR spectrum of the *n*-butanol extract of *C. majalis* containing isovitexin (**1**) and vicenin II (**2**).

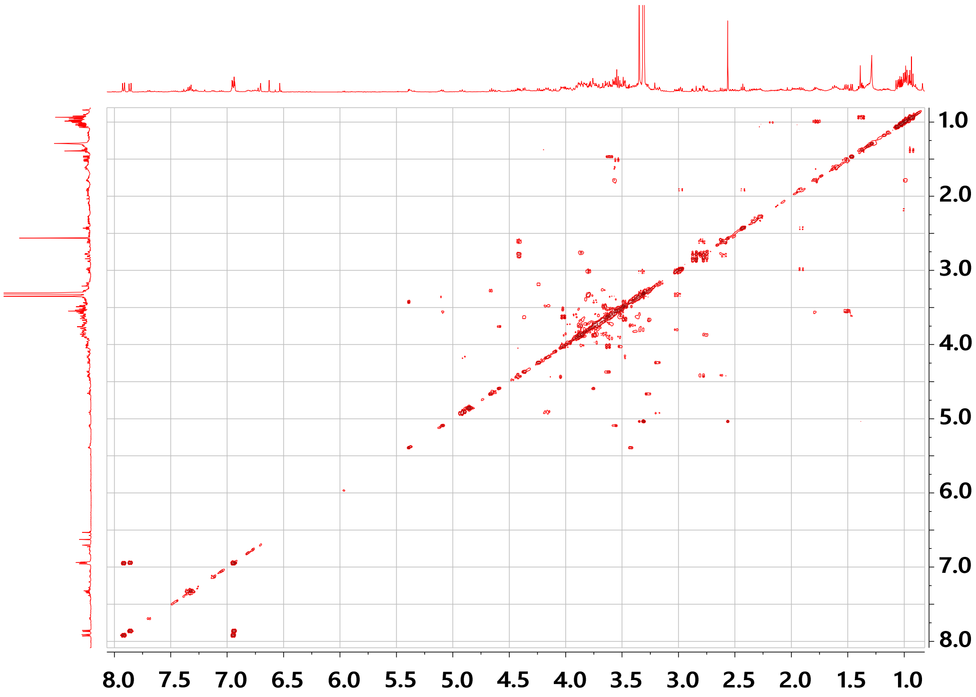

Figure S6. COSY spectrum of the *n*-butanol extract of *C. majalis* containing isovitexin (**1**) and vicenin II (**2**).

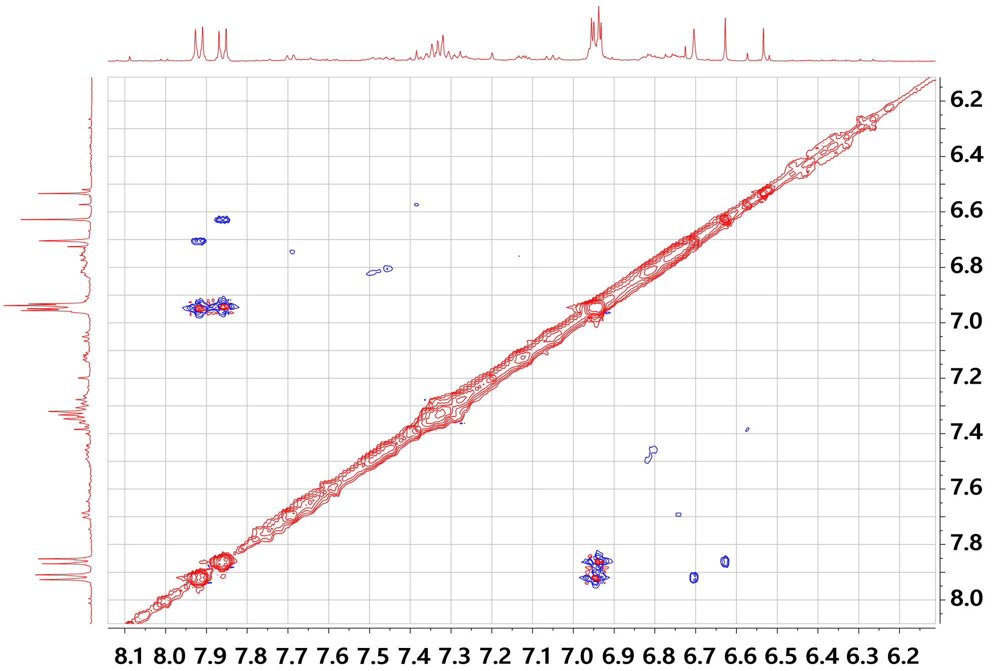

Figure S7. A part of NOESY spectrum of the *n*-butanol extract of *C. majalis* containing isovitexin (**1**) and vicenin II (**2**).

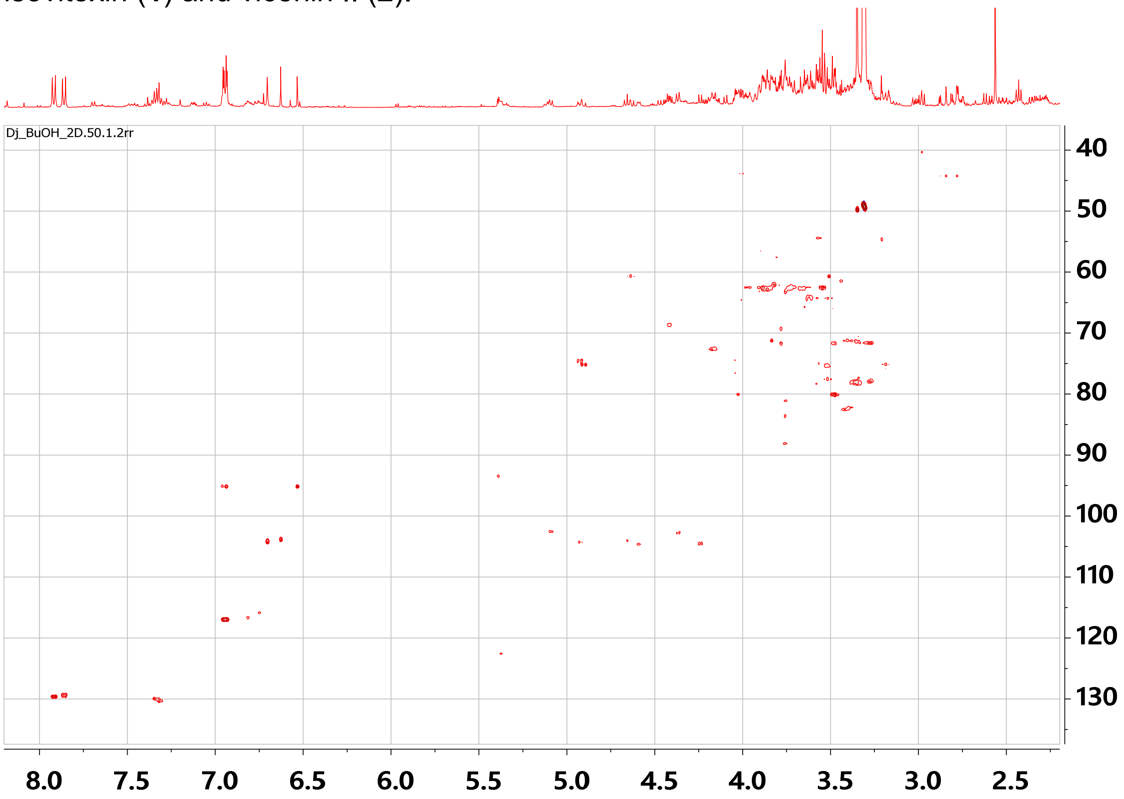

Figure S8. HSQC spectrum of the *n*-butanol extract of *C. majalis* containing isovitexin (**1**) and vicenin II (**2**).

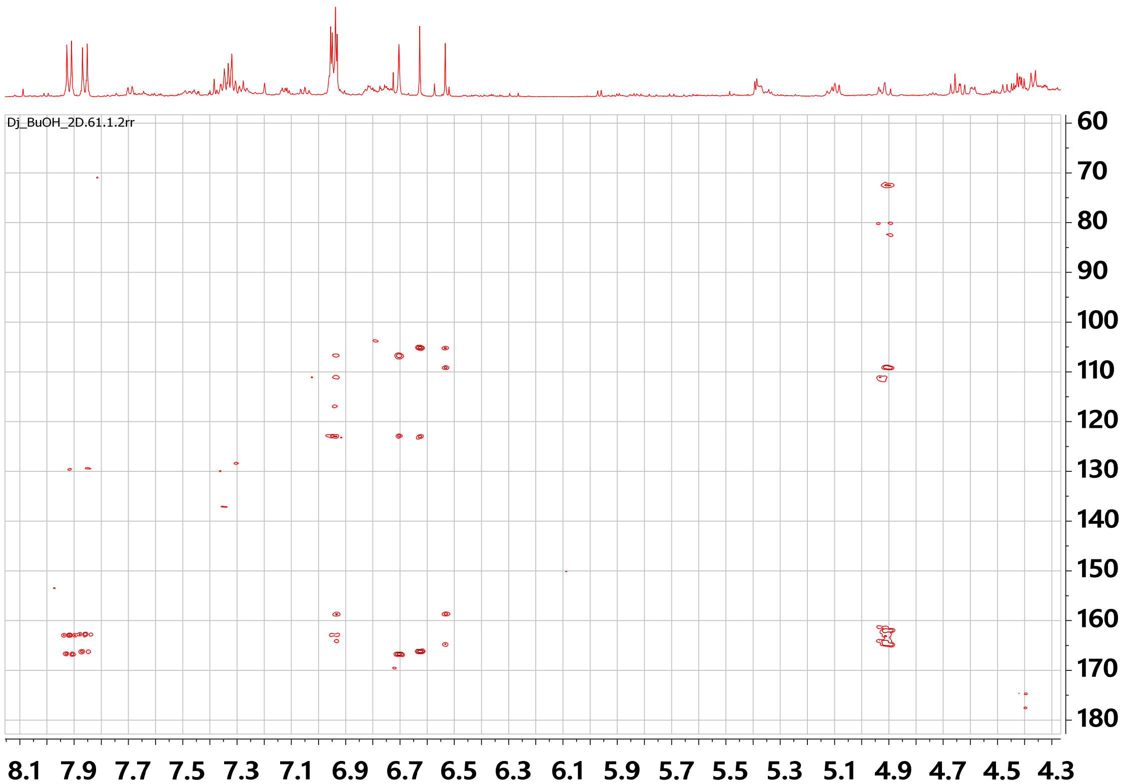

Figure S9. A part of HMBC spectrum of the *n*-butanol extract of *C. majalis* containing isovitexin (**1**) and vicenin II (**2**).

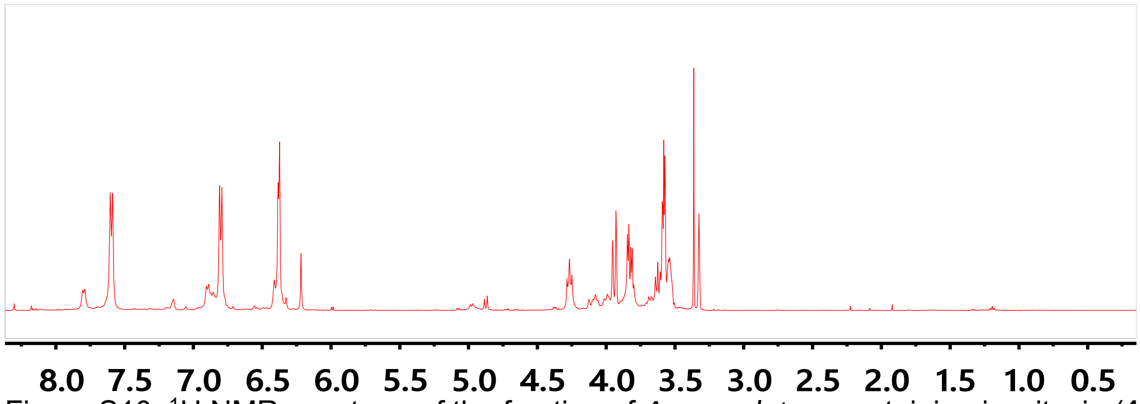

Figure S10. ^{1}H NMR spectrum of the fraction of *A. maculatum* containing isovitexin (**1**).

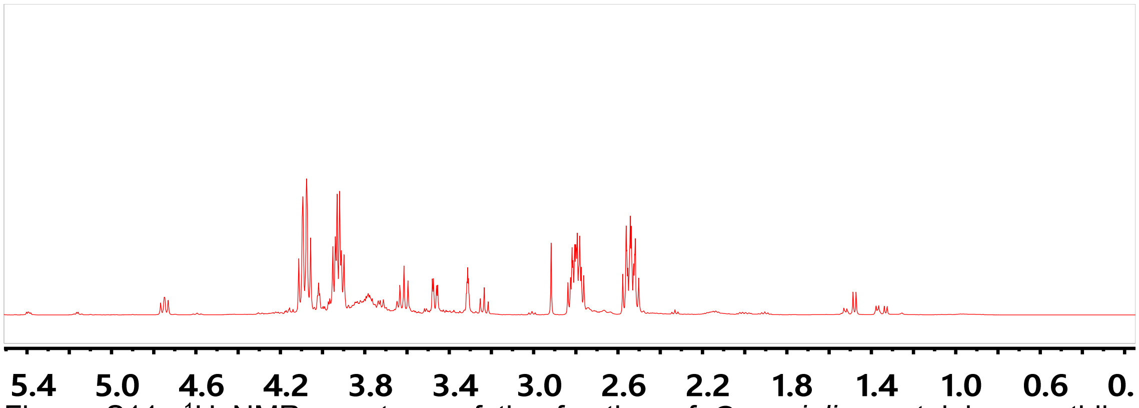

Figure S11. ^1H NMR spectrum of the fraction of *C. majalis* containing azetidine-2-carboxylic acid (**3**).

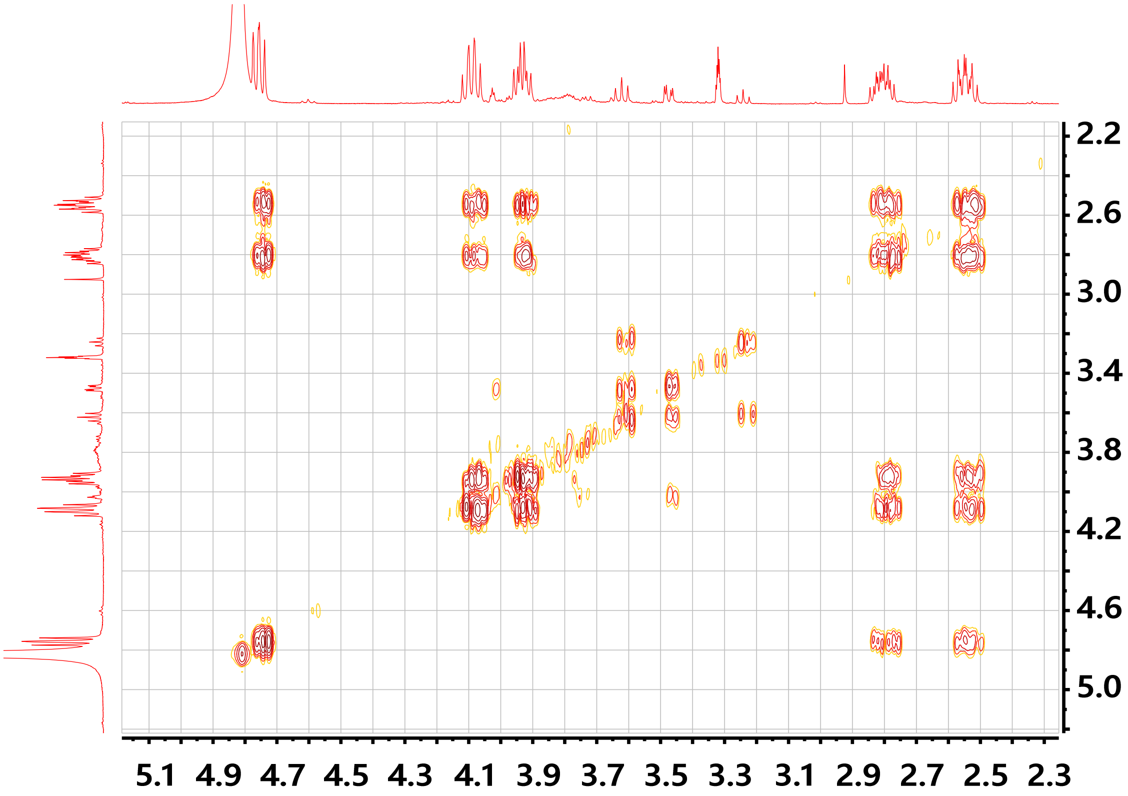

Figure S12. COSY spectrum of the fraction of *C. majalis* containing azetidine-2-carboxylic acid (**3**).

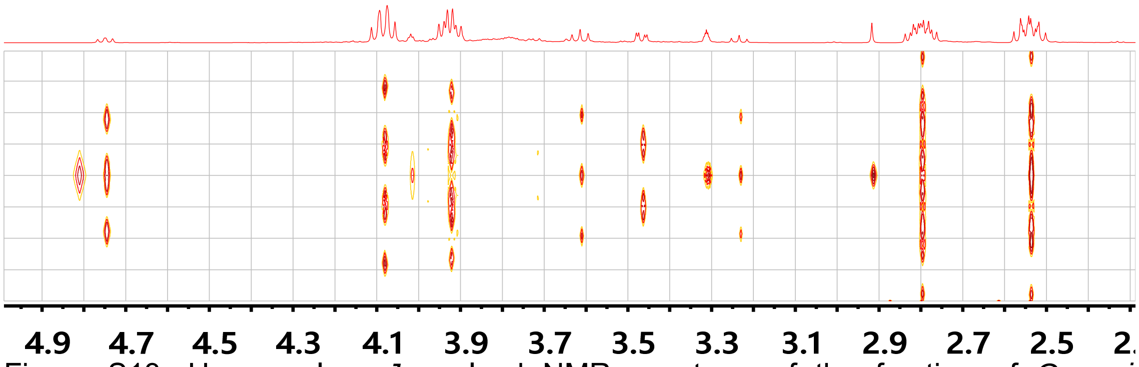

Figure S13. Homonuclear *J*-resolved NMR spectrum of the fraction of *C. majalis* containing azetidine-2-carboxylic acid (**3**).

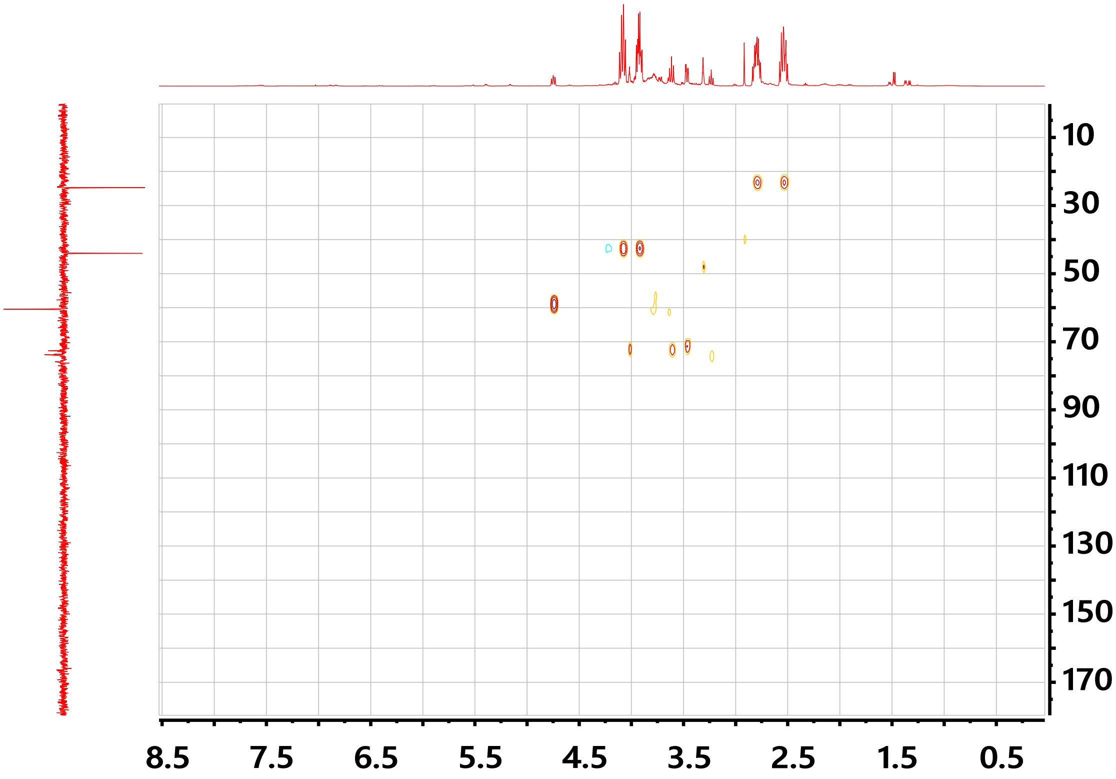

Figure S14. ^{1}H NMR spectrum of the fraction of *C. majalis* containing azetidine-2-carboxylic acid (**3**).

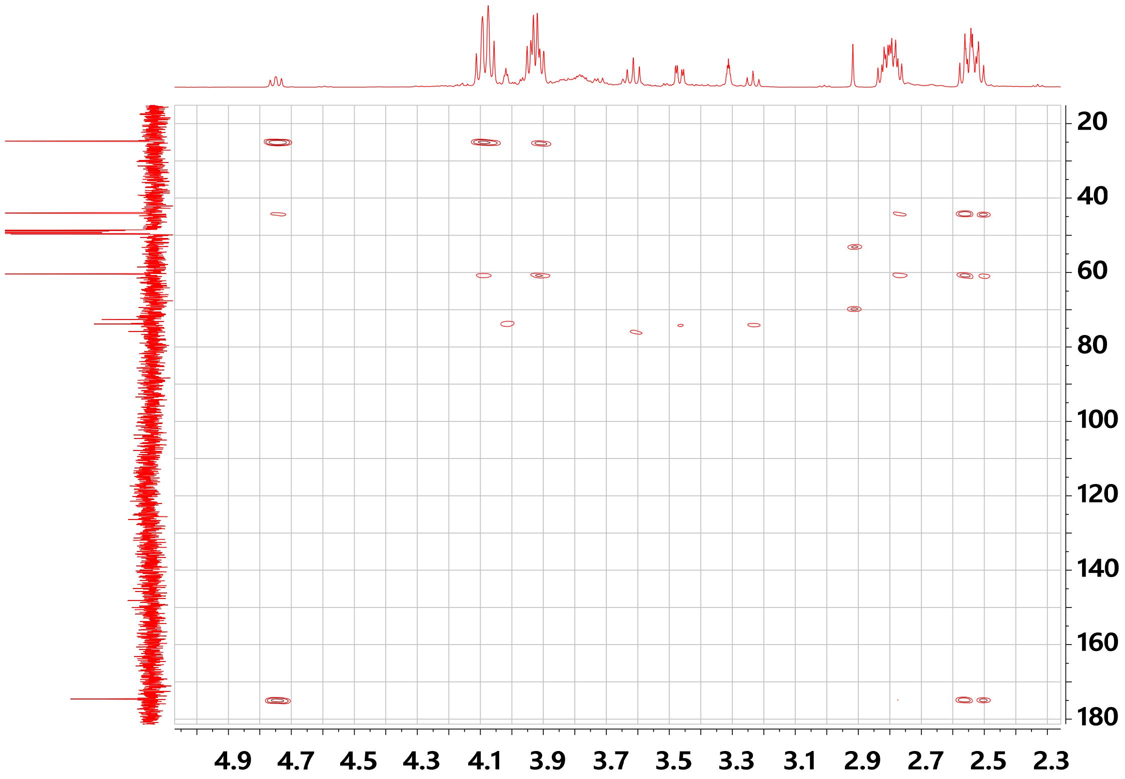

Figure S15. ^{1}H NMR spectrum of the fraction of *C. majalis* containing azetidine-2-carboxylic acid (**3**).

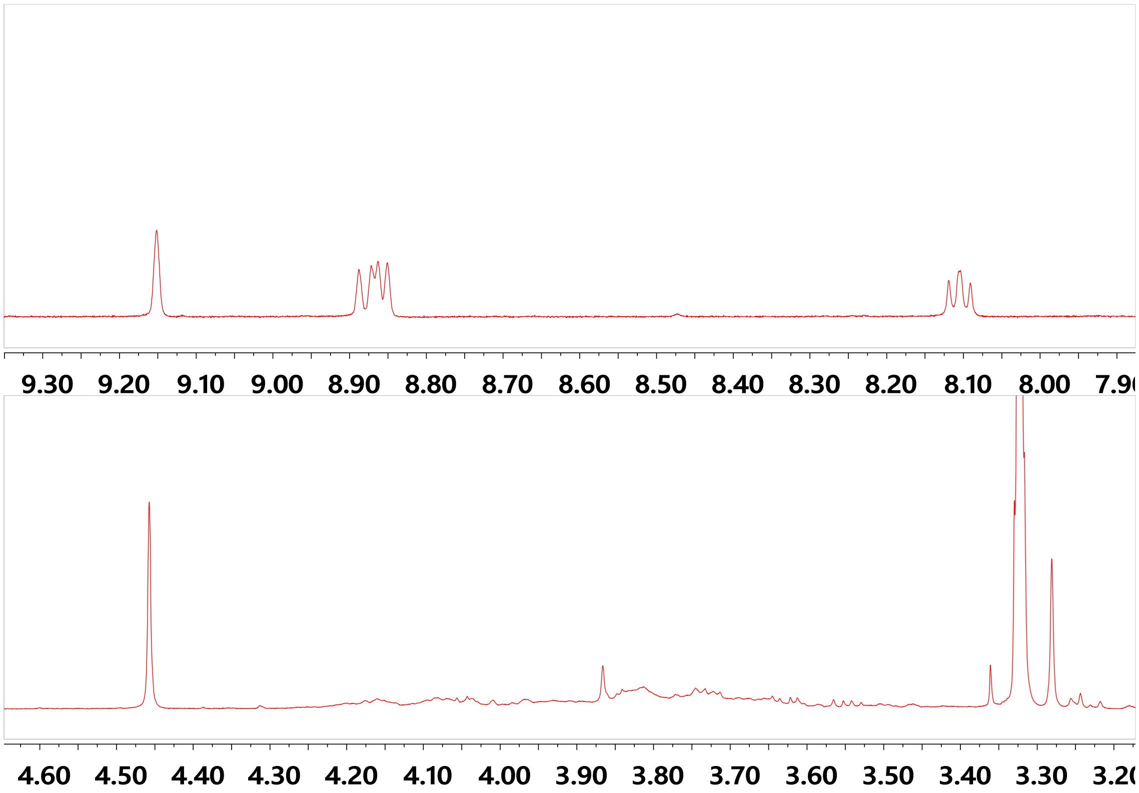

Figure S16. ^{1}H NMR spectrum of the fraction of *C. majalis* containing trigonelline (**4**).

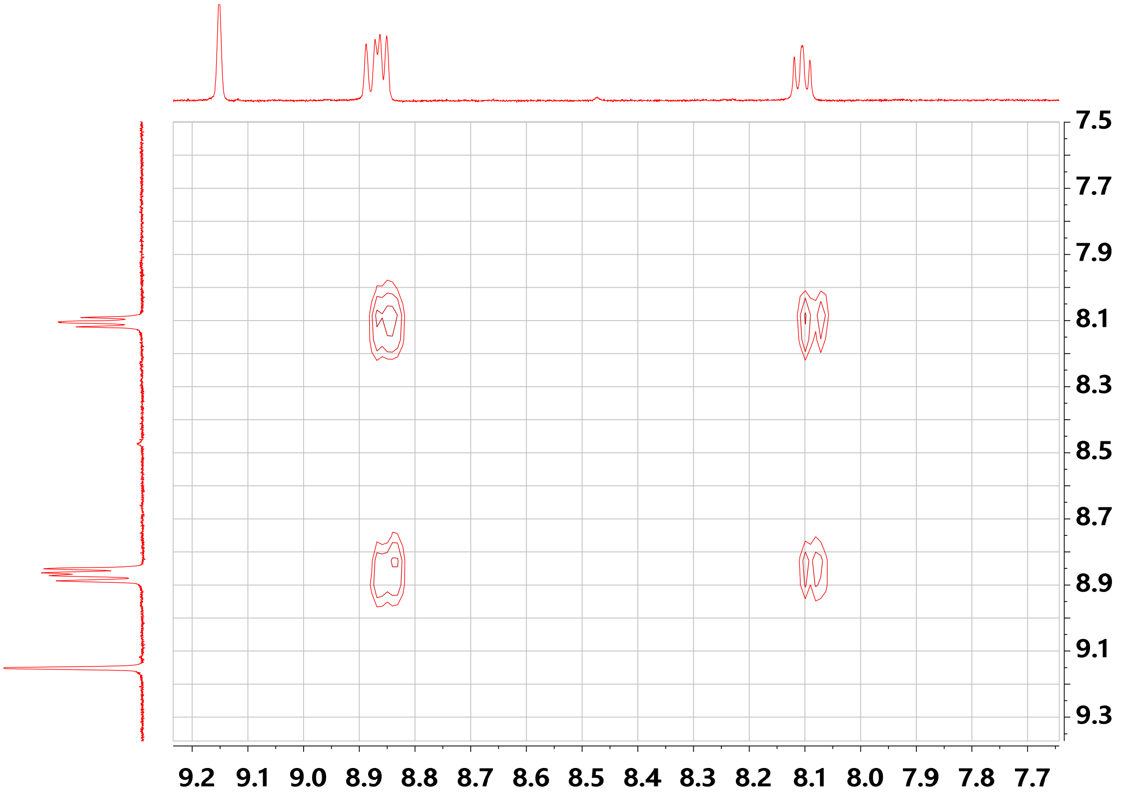

Figure S17. COSY spectrum of the fraction of *C. majalis* containing trigonelline (**4**).

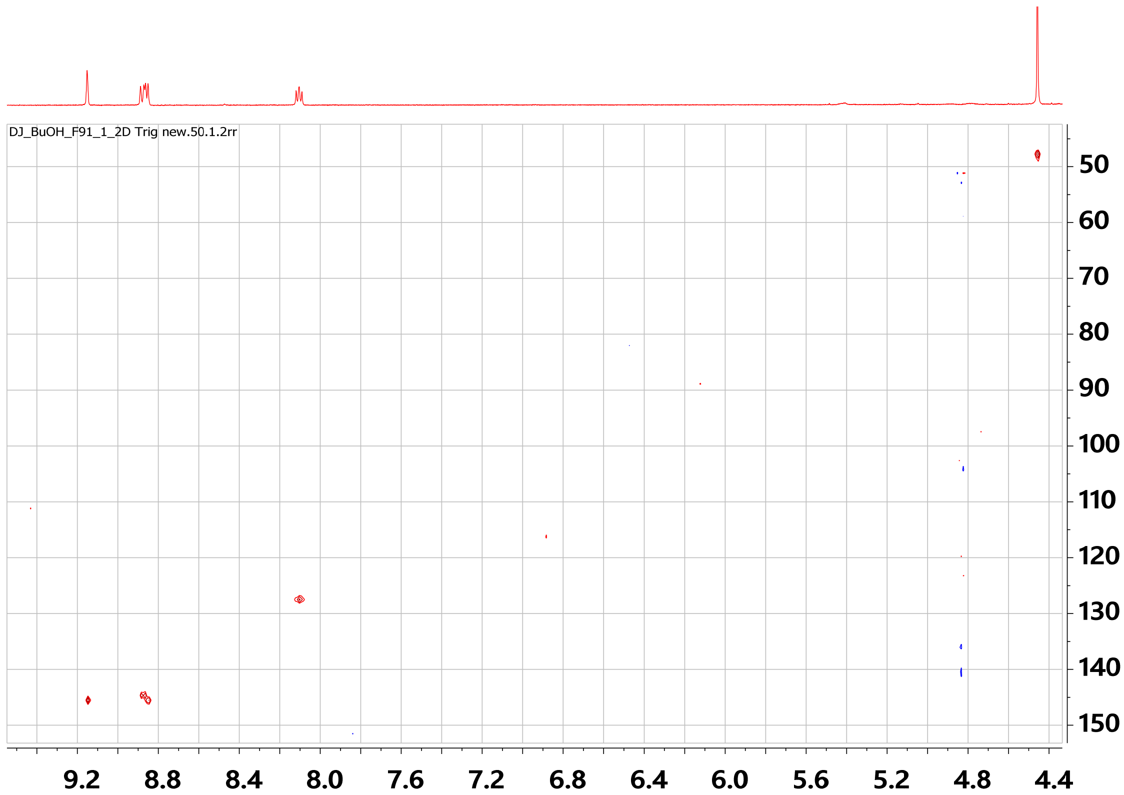

Figure S18. HSQC spectrum of the fraction of *C. majalis* containing trigonelline (**4**).

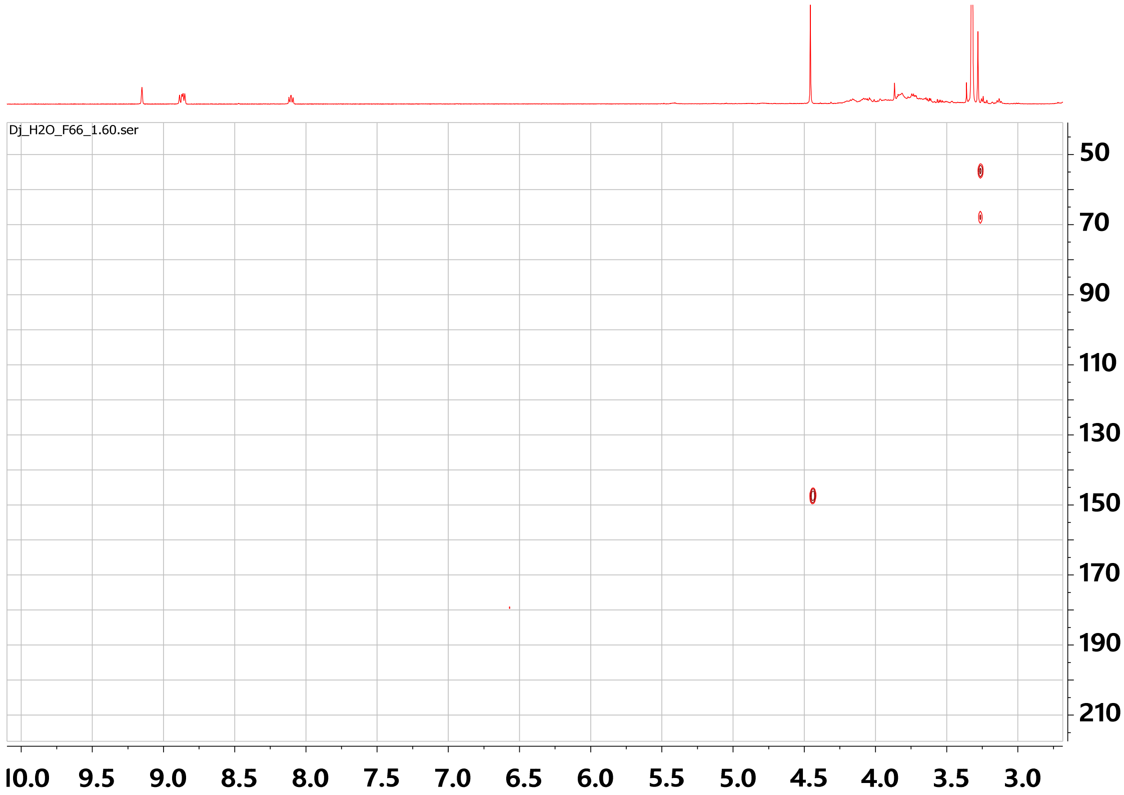

Figure S19. HMBC spectrum of the fraction of *C. majalis* containing trigonelline (**4**).

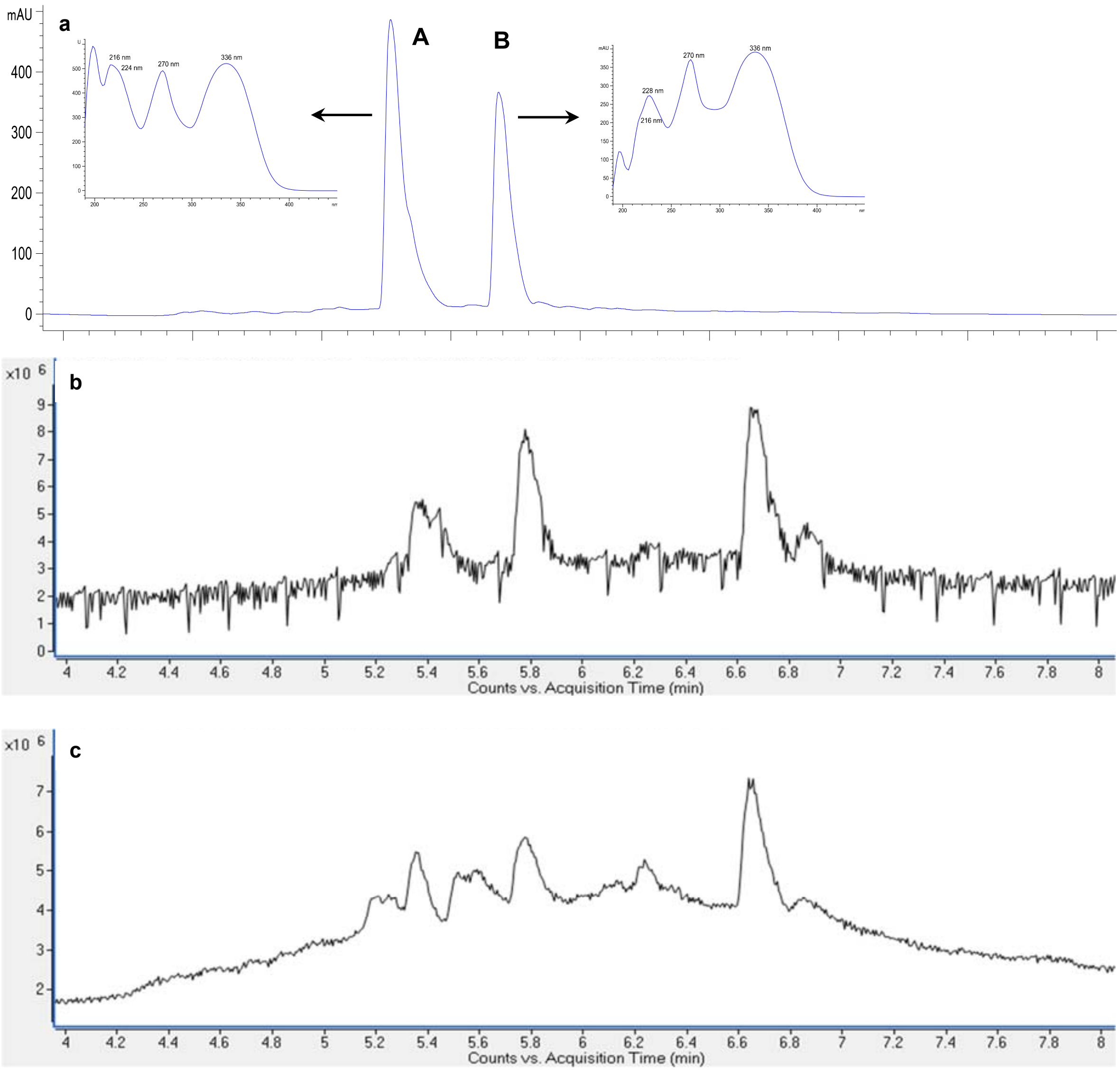

Figure S20. LC-DAD-MS analysis of the *C. majalis* extract: **a** LC-DAD (270 nm) chromatogram (4–8 min) and UV spectra of compounds **A** and **B**; **b** and **c** LC-ESI-MS total ion current (TIC) chromatograms (4–8 min) in negative and positive ionization modes, respectively.

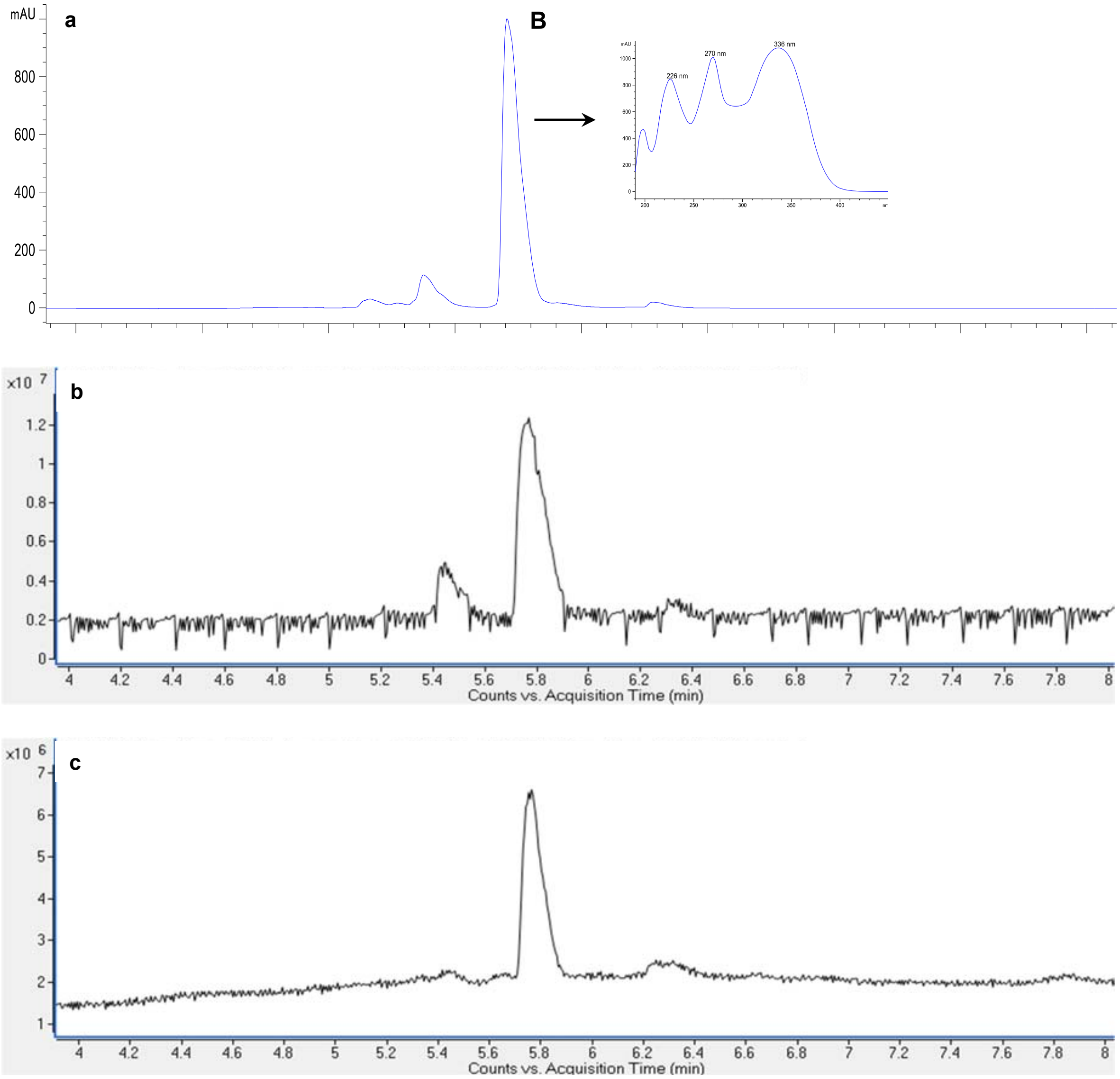

Figure S21. LC-DAD-MS analysis of the *A. maculatum* extract: **a** LC-DAD (270 nm) chromatogram (4–8 min) and UV spectrum of compound **B**; **b** and **c** LC-ESI-MS total ion current (TIC) chromatograms (4–8 min) in negative and positive ionization modes, respectively.

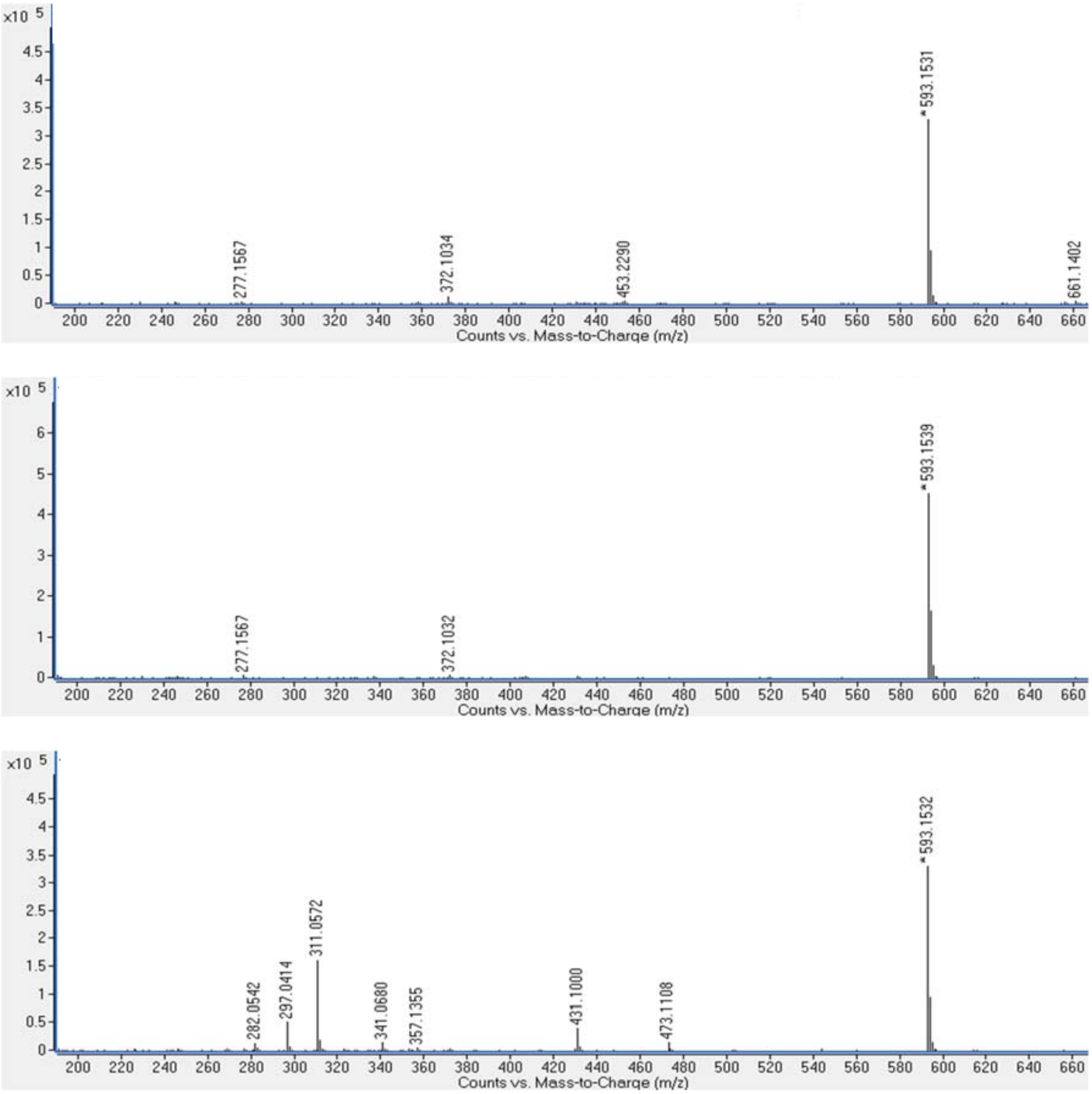

Figure S22. Negative mass spectra (*m/z* 200–660) of the compound **A** at CE = 0, 20 and 40 V, respectively (from top to the bottom) in the TIC chromatogram of the *C. majalis* extract.

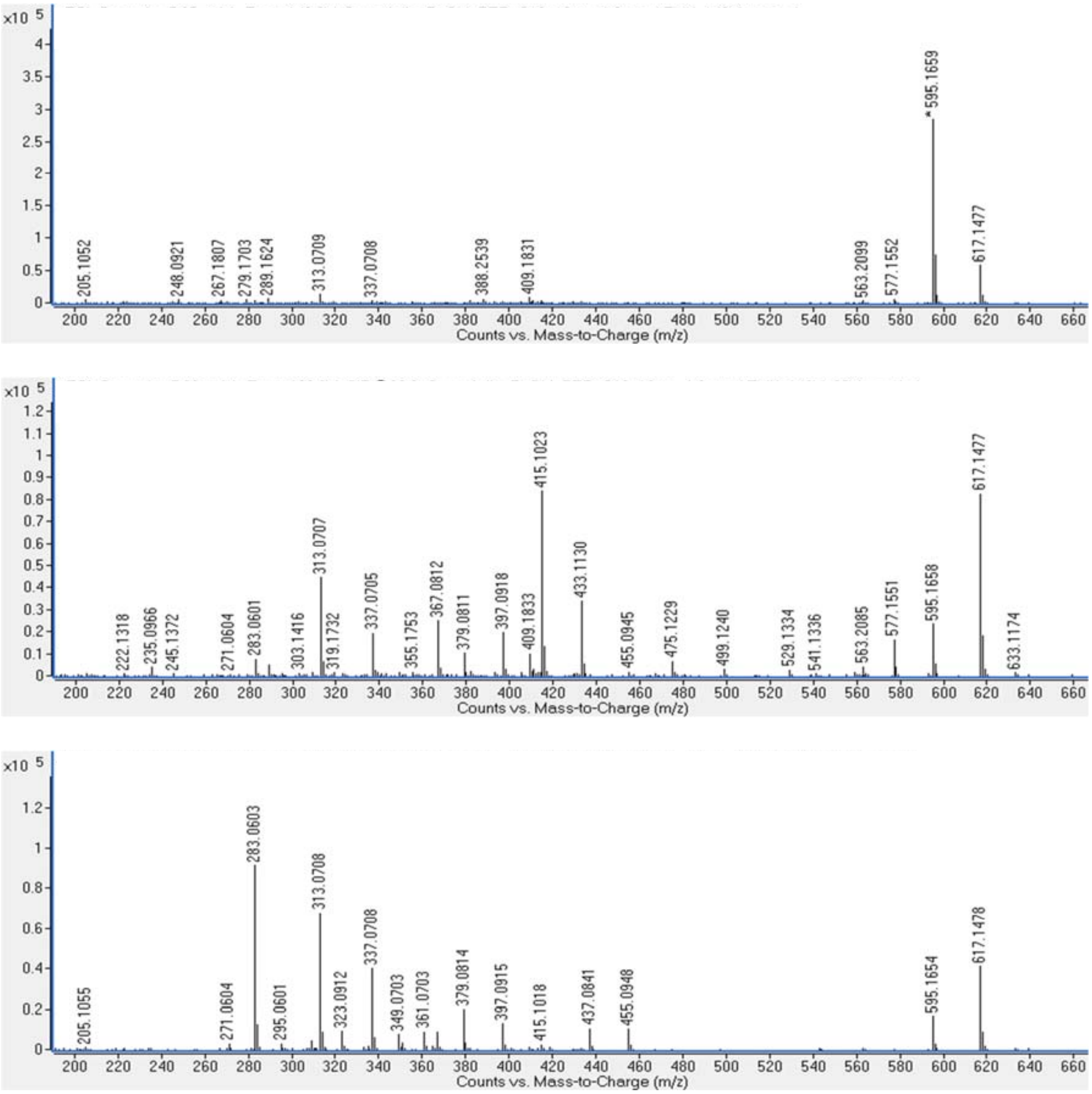

Figure S23. Positive mass spectra (*m/z* 200–660) of the compound **A** at CE = 0, 20 and 40 V, respectively (from top to the bottom) in the TIC chromatogram of the *C. majalis* extract.

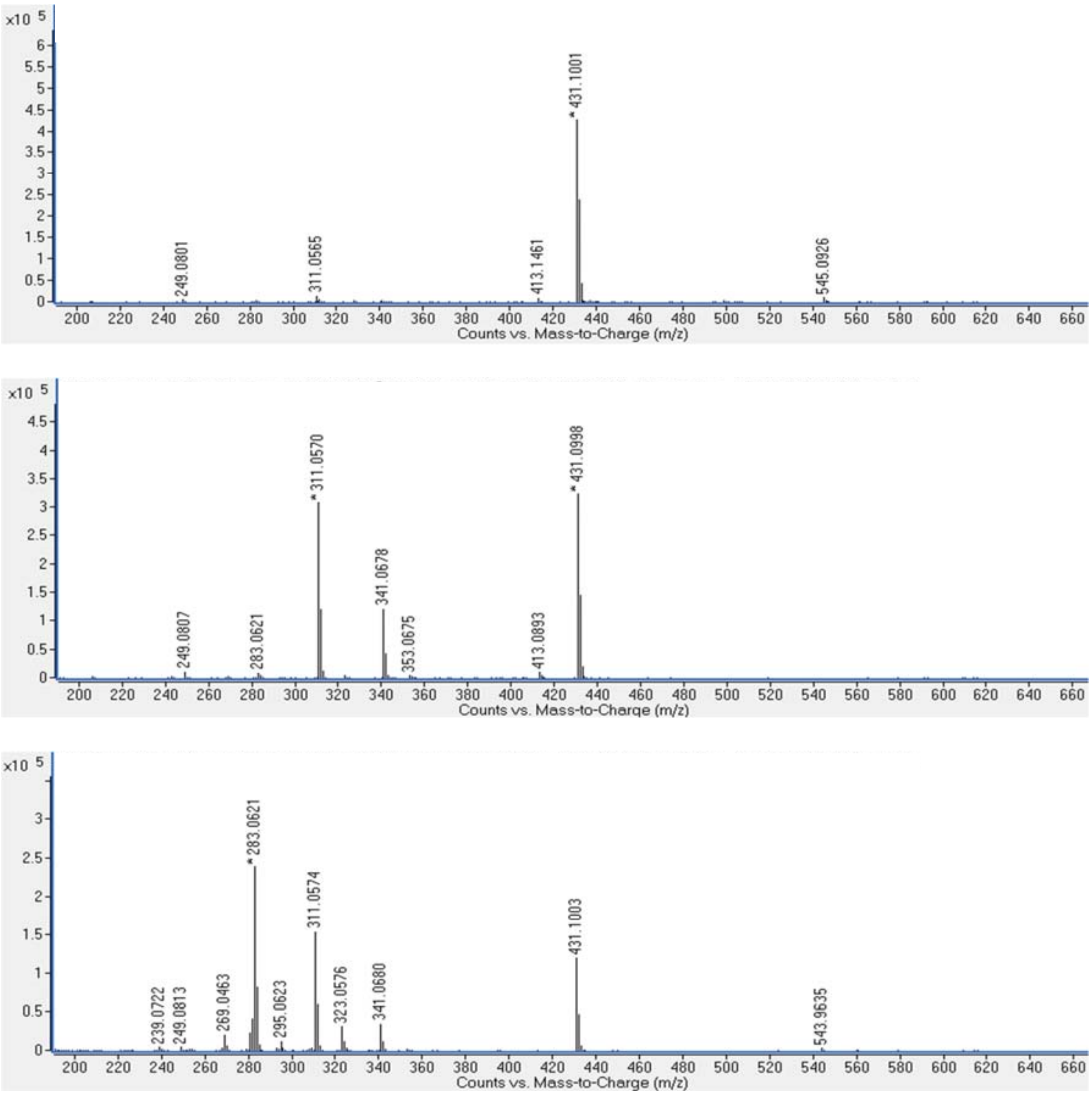

Figure S24. Negative mass spectra (*m/z* 200–660) of the compound **B** at CE = 0, 20 and 40 V, respectively (from top to the bottom) in the TIC chromatogram of the *C. majalis* extract.

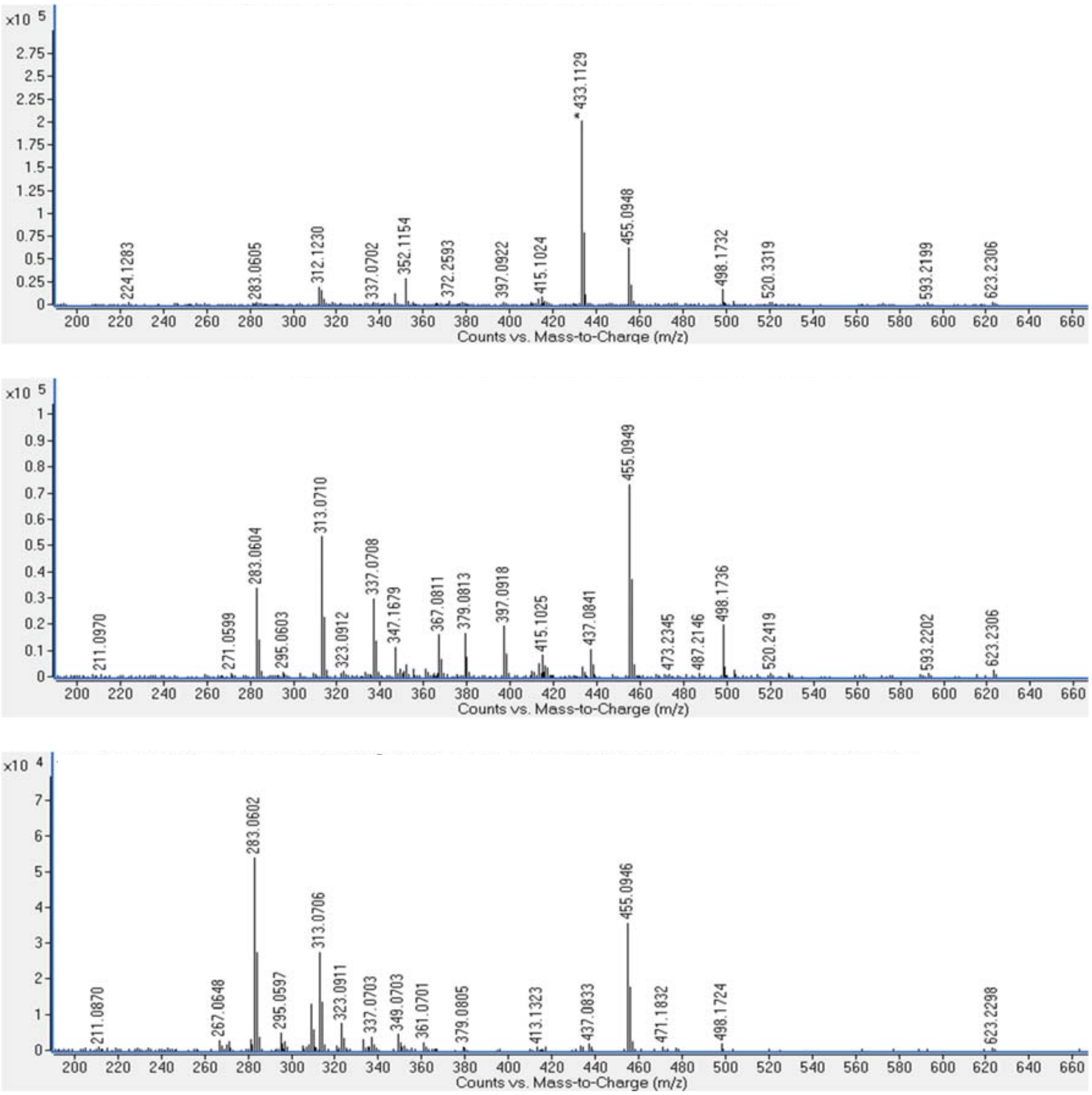

Figure S25. Positive mass spectra (*m/z* 200–660) of the compound **B** at CE = 0, 20 and 40 V, respectively (from top to the bottom) in the TIC chromatogram of the *C. majalis* extract.

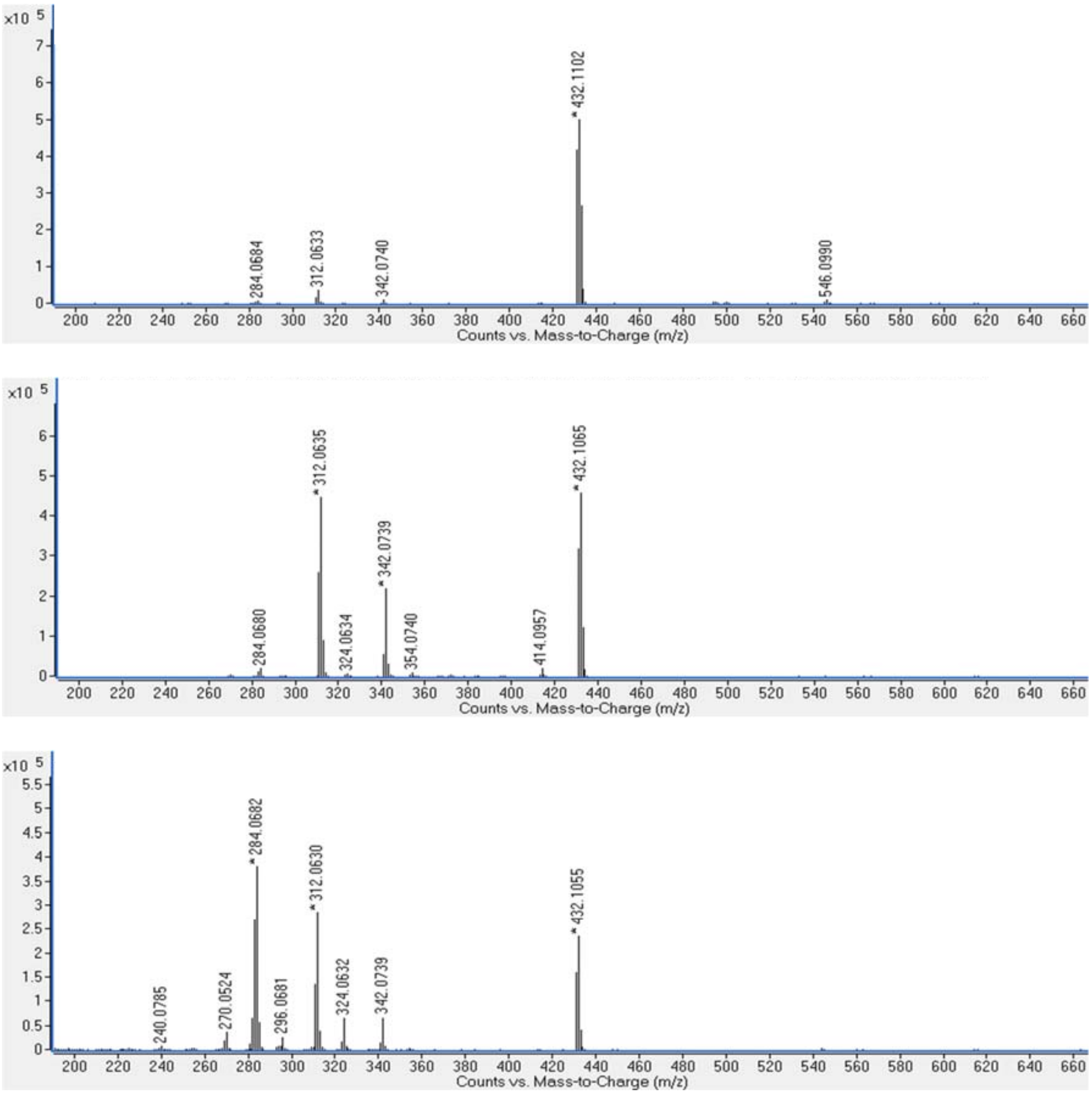

Figure S26. Negative mass spectra (*m/z* 200–660) of the compound **B** at CE = 0, 20 and 40 V, respectively (from top to the bottom) in the TIC chromatogram of the *A. maculatum* extract.

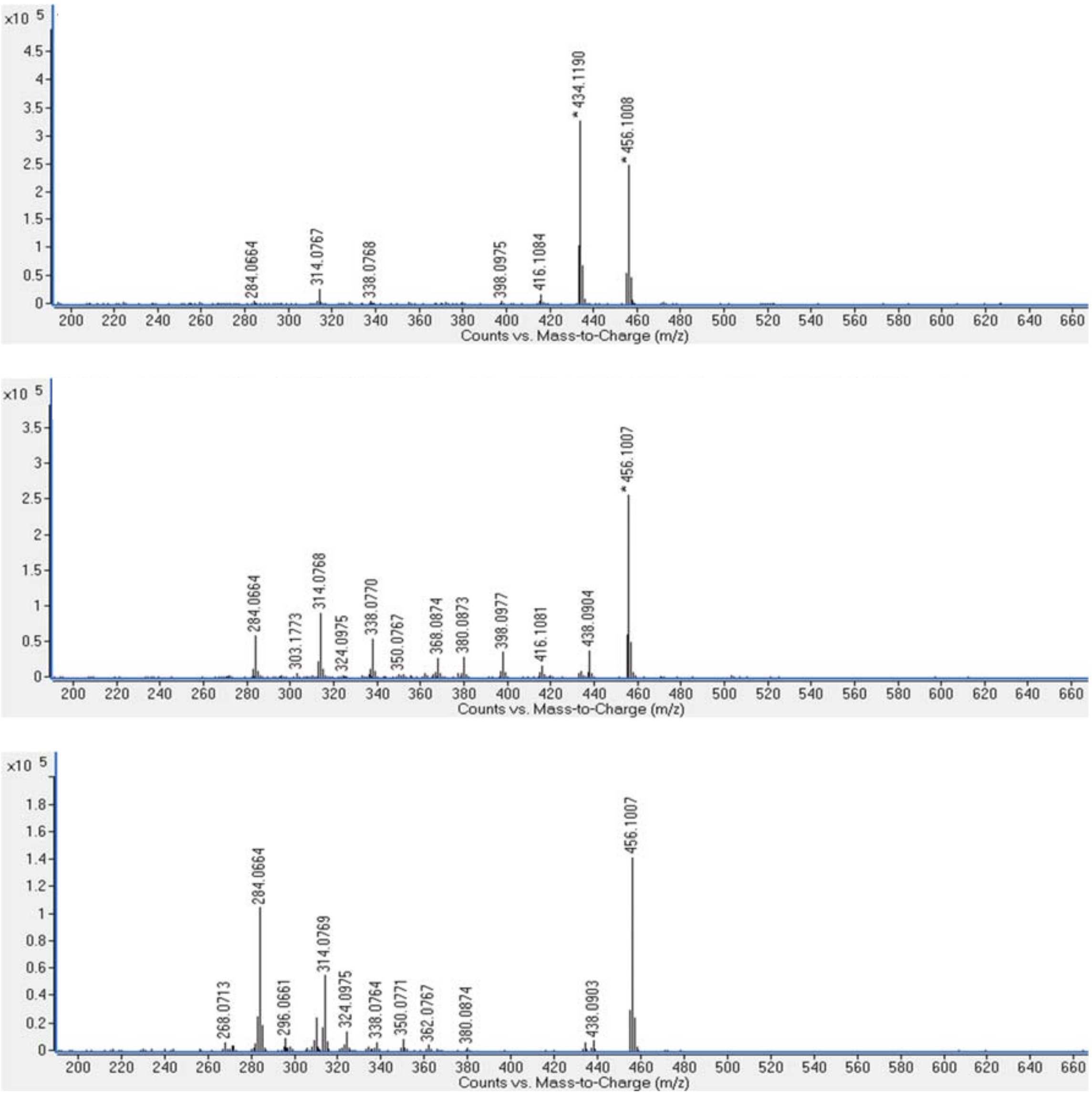

Figure S27. Positive mass spectra (*m/z* 200–660) of the compound **B** at CE = 0, 20 and 40 V, respectively (from top to the bottom) in the TIC chromatogram of the *A. maculatum* extract.
